# Effects of Mountain Uplift and Climatic Oscillations on Phylogeography and Species Divergence in Four Endangered *Notopterygium* Herbs

**DOI:** 10.3389/fpls.2017.01929

**Published:** 2017-11-08

**Authors:** Khurram Shahzad, Yun Jia, Fu-Lin Chen, Umar Zeb, Zhong-Hu Li

**Affiliations:** Key Laboratory of Resource Biology and Biotechnology in Western China, Ministry of Education, College of Life Sciences, Northwest University, Xi’an, China

**Keywords:** conservation, genetic structure, *Notopterygium*, phylogeography, Qinghai–Tibetan Plateau (QTP), species divergence

## Abstract

Mountain uplift and climatic fluctuations are important driving forces that have affected the geographic distribution and population dynamics history of organisms. However, it is unclear how geological and climatic events might have affected the phylogeographic history and species divergence in high-alpine herbal plants. In this study, we analyzed the population demographic history and species differentiation of four endangered *Notopterygium* herbs on the high-altitude Qinghai–Tibetan Plateau (QTP) and adjacent areas. We combined phylogeographic analysis with species distribution modeling to detect the genetic variations in four *Notopterygium* species (*N. incisum*, *N. franchetii*, *N. oviforme*, and *N. forrestii*). In total, 559 individuals from 74 populations of the four species were analyzed based on three maternally inherited chloroplast fragments (*matK*, *rbcL*, and *trn*S*-trn*G) and one nuclear DNA region (internal transcribed spacer, ITS). Fifty-five chloroplast DNA (cpDNA) and 48 ITS haplotypes were identified in the four species. All of the cpDNA and ITS haplotypes were species-specific, except *N. franchetii* and *N. oviforme* shared one cpDNA haplotype, H32. Phylogenetic analysis suggested that all four species formed a monophyletic clade with high bootstrap support, where *N. franchetii* and *N. oviforme* were sisters. In addition, each *Notopterygium* species generated an individual clade that corresponded to their respective species in the ITS tree. Population dynamics analyses and species distribution modeling showed that the two widely distributed herbs *N. incisum* and *N. franchetii* exhibited obvious demographic expansions during the Pleistocene ice ages. Molecular dating suggested that the divergence of the four *Notopterygium* species occurred approximately between 3.6 and 1.2 Mya, and it was significantly associated with recent extensive uplifts of the QTP. Our results support the hypothesis that mountain uplift and Quaternary climatic oscillations profoundly shaped the population genetic divergence and demographic dynamics of *Notopterygium* species. The findings of this and previous studies provide important insights into the effects of QTP uplifts and climatic changes on phylogeography and species differentiation in high altitude mountainous areas. Our results may also facilitate the conservation of endangered herbaceous medicinal plants in the genus *Notopterygium.*

## Introduction

Geological events and climatic fluctuations are considered to have profoundly shaped the distribution and population dynamics history of species in mountain areas (Hewitt, 2004; Hickerson et al., 2010). Thus, during glacial periods, most species experienced adverse weather conditions in high altitude mountains, where they contracted into refugia in low latitudes and then their ranges expanded again after the ice ages, thereby leading to species divergence or secondary contact evolution (Hewitt, 2004; Nybom, 2004; Ohsawa and Ide, 2008). In addition, the distribution patterns and population genetic structures of some species were reshaped due to geographic barriers and climatic oscillations. Moreover, the population size, mating system, and bio-characteristics of species had important effects on the divergence and evolutionary history of populations of species (Stewart et al., 2010; Zhang et al., 2015). For example, some studies have suggested that the locations of ice age refugia for plants were determined mainly by the adaptability of species to the external environment (Stewart et al., 2010; Liao et al., 2015; Wang et al., 2015).

During the Quaternary ice periods, many species experienced extinction events due to repeated bottlenecks and genetic drift, which led to further divergent evolution and genetic isolation within species (Soltis et al., 2006; Hickerson et al., 2010; Stewart et al., 2010; Keppel et al., 2012). Repeated environmental changes may also have promoted the fragmentation of habitats, as well as causing exotic distributions of different species or intraspecific genetic changes (Hickerson et al., 2010; Jia et al., 2012). Studies of alpine trees have shown that populations were crossed during mountain uplift processes, whereas the exchange of genes among populations was restricted due to climatic and geographic barriers (McLachlan et al., 2005; Soltis et al., 2006; Birks and Willis, 2008; Avise, 2009; Gao et al., 2012). Some alpine species experienced deep lineage divergence due to climatic changes and environmental isolations (Qiu et al., 2011; Liu et al., 2012; Xu et al., 2015).

In the high latitudes of Europe and North America, studies suggested that plant species could have survived in high elevation areas (“invisible refugia”) during the ice age periods (Anderson et al., 2006; Provan and Bennett, 2008; Parducci et al., 2012; Stewart and Stringer, 2012; Ortego et al., 2012, 2015; Guichoux et al., 2013; Allen et al., 2015; Cavender-Bares et al., 2015). The presence of *Juniperus* species in the Qinghai-Tibetan Plateau (QTP) region also supports the existence of invisible refugia (Opgenoorth et al., 2010). The QTP is the largest and highest plateau in the world, with a mean altitude of more than 4000 m. Various endangered species and high levels of global diversity are present on this plateau (Mittermeier et al., 2005). Studies suggest that extensive uplifts of the QTP occurred in the Miocene–Pliocene era between 3.6 and 1.7 Mya (Li and Fang, 1999; Zhang et al., 2000; Zhou et al., 2006). The lifting of mountains triggered species divergence and changed the genetic structure to affect the evolution of high-alpine plants (Liu et al., 2012; Wen et al., 2014; Ickert-Bond and Renner, 2016). In particular, the geological effects of the QTP on the genetic structure, geographic distribution, and species differentiation of plants have been clearly defined in this area (Wang et al., 2010; Xu et al., 2010; Li et al., 2013; Liu et al., 2013; Sun et al., 2014; Favre et al., 2015; Hughes and Atchison, 2015). However, most of these previous studies focused on the response patterns of tree or shrub species to mountain uplifts and climatic oscillations on the QTP (Liu et al., 2006; Wang et al., 2009; Mao et al., 2010; Xu et al., 2010; Tian et al., 2011; Qiu et al., 2011; Wen et al., 2014; Favre et al., 2015; Hughes and Atchison, 2015), whereas little is known about the effects of mountain uplifts and climate events on cold-tolerant herbal species in the high altitude QTP and adjacent regions.

The genus *Notopterygium* H. de Boissieu (Apiaceae) comprises perennial and endangered herbaceous medicinal plants, which are mainly distributed in the QTP and its surrounding high-altitude areas. According to records in the *Flora of China*, this genus comprises six species: *N. incisum* C. C. Ting ex H. T. Chang, *N. oviforme* R. H. Shan, *N. franchetii* H. de Boissieu, *N. forrestii* H. Wolff, *N. tenuifolium* M. L. Sheh and F. T. Pu, and *N. pinnatiinvolucellum* F. T. Pu and Y. P. Wang. *N. incisum* and *N. franchetii* have wide distribution ranges at altitudes of 3200–5100 m and 1700–4500 m, respectively. *N. oviforme* occurs in the eastern part of the QTP at altitudes of 1700–3200 m. The other three species, i.e., *N. forrestii* (4000–4300 m), *N. tenuifolium* (4300 m), and *N. pinnatiinvolucellum* (3400 m), have very limited distributions among the high-alpine shrubs and meadows in the west region of China. These herb species provide an excellent model for detecting the effects of the QTP uplifts and Quaternary climatic oscillations on the genetic structure and species divergence of plants. However, in recent years, due to high market demand, the wild resources of these *Notopterygium* species have decreased rapidly because of human over-exploitation (Zhou et al., 2010). The *Notopterygium* species are now listed as endangered herb species in the IUCN Red List, and their management and conservation are urgently required (Wu et al., 2005). Information regarding geographic distributions and genetic diversity is vital for formulating effective conservation strategies for wild plant resources. However, most of the previous studies of the *Notopterygium* species have focused mainly on their phylogenetic evolutionary relationships (Pu et al., 2000; Yang et al., 2017), morphological and physiological characteristics (She and Pu, 1996; Wang et al., 1996; Jiang et al., 2005), and comparative transcriptome analysis (Jia et al., 2017), whereas little is known about their genetic divergence and population demographic history.

In the current study, we sampled four species, i.e., *N. incisum*, *N. oviforme*, *N. franchetii*, and *N. forrestii*, across their entire geographic distributions in the high-altitude QTP and adjacent areas. We detected the genetic variations in three chloroplast DNA (cpDNA) markers and a nuclear DNA fragment in order to characterize the population histories and species divergence of these endangered herb plants. Our aims were: (1) to determine the genetic structure and population evolutionary history of four *Notopterygium* species; (2) to identify the phylogenetic relationships among these species and their phylogeographic history; (3) to explore the effects of QTP uplifts and climatic changes in the Quaternary on the divergence and phylogeography of these species; and (4) to propose reasonable conservation and management strategies for the endangered *Notopterygium* species.

## Materials and Methods

### Sample Collection

**FIGURE 1:**
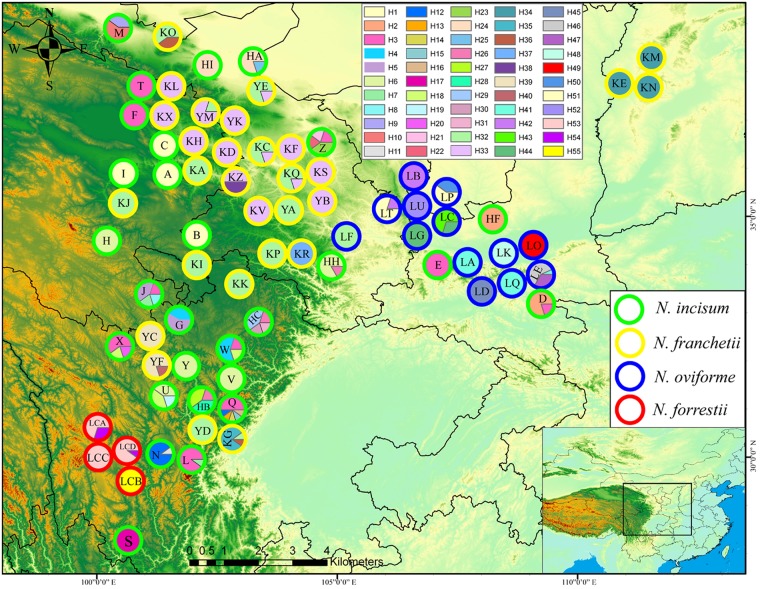
Geographic distribution of cpDNA haplotypes for the four *Notopterygium* species. Each circle represents a population and each color represents each haplotype. The colored outlines of the circles distinguish the four species, where green indicates *N. incisum*, yellow indicates *N. franchetii*, blue indicates *N. oviforme*, and red indicates *N. forrestii*.

In this study, in order to obtain information about genetic variation over a wide area, 559 individuals from 74 populations were collected for the four *Notopterygium* species in Sichuan, Shaanxi, Gansu, Qinghai, and Shanxi provinces in the high-altitude QTP and adjacent areas. These samples covered the complete geographic distribution ranges of the four species in the QTP and surrounding areas. From 2 to 17 individuals were sampled from each population, where all of the samples collected were separated from each other by at least 100 m. Detailed information about the latitude, longitude, and altitude for all of the populations is provided in **Figure 1** and **Table 1**. All of the materials and documents have been deposited in the College of Life Sciences, Northwest University. In addition, two species from the genus *Pleurospermum*, i.e., *P. prattii* and *P. franchetianum*, as well as *Heracleum moellendorffii* were used as outgroups.

**Table 1:** Geographic distributions of the four *Notopterygium* species sampled in this study.

Species	Population	Sample	Location	Longitude	Latitude	Altitude (m)
*N. incisum*	A	13	Huzhubeishan, Qinghai	102.4319	36.8918	2620
	B	5	Maqu, Gansu	102.0703	33.9992	3479
	C	10	Datong, Qinghai	101.8527	37.1496	3030
	D	10	Taibaishan, Shaanxi	108.7797	33.8532	2800
	E	5	Guangtoushan, Shaanxi	107.7010	34.0535	3190
	F	10	Qinglinxiang, Qinghai	101.4009	37.1007	2756
	G	12	Jiuzhi, Qinghai	101.6890	32.8584	4030
	H	10	Maqin, Qinghai	100.1971	34.4904	4030
	I	10	Tongde, Qinghai	100.5467	35.2760	3259
	J	10	Aba, Sichuan	101.0998	33.3834	4030
	L	10	Kangding, Sichuan	101.9669	29.9889	3560
	M	10	Zhangye, Gansu	100.4498	38.9259	3100
	N	10	Yajiang, Sichuan	101.3272	30.0611	3540
	Q	10	Baoxing, Sichuan	102.8176	30.3683	3442
	S	10	Muli, Sichuan	100.6510	28.2637	3750
	T	5	Qinglinxiang, Qinghai	101.5308	37.3207	3200
	U	6	Daofu, Sichuan	101.3826	31.4693	3920
	V	6	Xiaojin, Sichuan	102.6387	32.1214	3219
	W	6	Xiaojin, Sichuan	102.7960	32.2396	3900
	X	8	Ganzi, Sichuan	100.4784	32.3009	4073
	Y	2	Luhuo, Sichuan	101.5595	31.8943	3465
	Z	5	Yuzhong, Gansu	104.3608	35.7666	3046
	HA	5	Tianzhu, Gansu	103.2542	37.9120	3102
	HB	5	Danba, Sichuan	102.1852	30.9335	3708
	HC	5	Barkam, Sichuan	103.3875	32.7876	4652
	HF	5	Taibai, Shaanxi	108.2254	34.9387	3323
	HH	6	Zhouqu, Gansu	104.5106	34.1207	3360
	HI	5	Datong, Qinghai	102.2978	38.1388	3150
*N. franchetii*	KA	9	Huzhubeishan, Qinghai	102.4319	36.8918	2110
	KC	10	Heping, Gansu	103.9551	36.0039	2450
	KD	10	Qilisi, Qinghai	102.7054	36.0847	2450
	KE	2	Jiaocheng, Shanxi	111.4510	37.7604	2750
	KF	7	Xinglongshan, Gansu	104.0576	35.7966	2484
	KG	10	Ya’an, Sichuan	102.8176	30.3683	2890
	KH	10	Datong, Qinghai	101.8527	37.1496	2319
	KI	9	Maqu, Gansu	102.0703	33.9992	2379
	KJ	10	Tongde, Qinghai	100.5467	35.2760	2273
	KK	10	Nuoergai, Sichuan	102.9615	33.5903	3526
	KL	10	Datong, Qinghai	101.5308	37.3207	3200
	KM	10	Yundingshan, Shanxi	111.5310	37.8906	2543
	KN	2	Jiaocheng, Shanxi	111.4852	37.6826	2622
	KO	5	Zhangye, Gansu	101.4667	38.7167	2800
	KP	5	Yanchang, Gansu	104.2480	34.2263	2520
	KQ	10	Xinglongshan, Gansu	104.0375	35.7778	2400
	KR	2	Yanchang, Gansu	104.2590	34.2257	2470
	KS	5	Lintao, Gansu	103.8596	35.3950	1883
	KV	6	Hezheng, Gansu	103.3487	35.4249	2143
	KX	6	Qinglinxiang, Qinghai	101.4009	37.0841	2058
	KZ	6	Jishishan, Gansu	102.8741	35.7181	2281
	YA	5	Weiyuan, Sichuan	103.9837	35.1236	1760
	YB	5	Yuzhong, Gansu	104.6744	35.3104	2847
	YC	9	Daofu, Sichuan	101.3203	31.8562	3189
	YD	5	Danba, Sichuan	102.2000	30.5666	3318
	YE	6	Wuwei, Tianzhu, Gansu	103.4026	37.5991	2816
	YF	6	Luhuo, Sichuan	101.2372	31.8868	3246
	YK	5	Datong, Qinghai	102.3505	37.1864	3058
	YM	5	Foshan Forest Farm, Qinghai	102.2592	37.1722	2958
*N. oviforme*	LA	10	Taibaishan, Shaanxi	107.7011	34.0535	3190
	LB	10	Huating, Gansu	106.5856	35.1610	2650
	LC	10	Long, Shaanxi	106.6734	35.0690	2568
	LD	17	Zhuque Forest Park, Shaanxi	108.5268	33.9248	1890
	LE	15	Chanan, Shaanxi	108.8230	33.8205	2430
	LF	10	Gangu, Gansu	105.1848	34.5744	2234
	LG	2	Xihuazhen, Gansu	106.5821	35.1609	2480
	LK	5	Meiyukou, Shaanxi	108.7230	33.7205	2300
	LO	5	Ningshan, Xunyang, Shaanxi	109.0716	34.4094	2410
	LP	5	Longxian, Guanshan, Shaanxi	107.1760	35.5032	2153
	LQ	5	Feng yukou, Shaanxi	108.6230	33.6205	2100
	LT	5	Hua, Gansu	106.4023	35.1588	2230
	LU	6	Hua, Gansu	106.6531	35.2182	2120
*N. forrestii*	LCA	10	Yajiang, Sichuan	100.5662	30.1583	4164
	LCB	10	Yajiang, Sichuan	100.7859	30.0441	4220
	LCC	10	Litang, Sichuan	100.3092	29.9981	4010
	LCD	10	Cara Mountain, Sichuan	100.6326	30.1369	4300


### DNA Extraction and Sequencing

Total DNA was extracted using the modified CTAB method (Doyle and Doyle, 1987) or with a plant DNA extraction kit (Tiangen, Beijing, China). We used 1% agarose gels to check the quality of the DNA extracted from the *Notopterygium* species. To screen for suitable primers, we first randomly selected 50 individuals (one individual from each population) to amplify the universal cpDNA primers and nDNA primers recommended by the Consortium for the Barcode of Life (CBOL) (CBOL Plant Working Group, 2009). Finally, three highly variable cpDNA primers, i.e., *trn*S-*trn*G, *matK*, and *rbcL*, and one nDNA internal transcribed spacer (ITS) primer were selected to determine the genetic variations in the genus *Notopterygium* after initial tests with six loci (Supplementary Table S1). Thus, two monomorphic cpDNA loci (*trnL-trnF* and *rpl36-infA*; Bai et al., 2010; Soumaya et al., 2014) were excluded from all of the subsequent analyses.

PCR amplification was performed in a volume of 25 μL containing 2 μL DNA template (10–50 ng/μL), 12.5 μL PCR MIX (Xi’an Runde, China), 0.75 μL of each primer (20 ng/μL), and 9 μL double-distilled H_2_O. The PCR reaction conditions were as described in Supplementary Table S2. All of the high quality PCR products were sequenced using the amplified forward and reverse primers with an ABI 3730 XL genetic analyzer (Applied Biosystems, Foster City, CA, United States). All of the sequences were deposited in GenBank under accession numbers MF787385–MF787579.

### Proofreading and Alignment of DNA, and Data Analysis

BioEdit v 7.0.9.0 (Hall, 1999) software was used for manual proofreading and checking the variable sites. MEGA v 7.0 (Sudhir et al., 2008) was used to remove low quality sequences and only high quality sequences were analyzed. For the ITS sequences, we visualized the possible color spectrum of the overlapping peaks at any one variable site. If a strong signal peak was more than half of a weak signal peak, then we used the strong peak for phrasing. If both the peaks overlapped, we used the following phrases for each variable site instead of both peaks as phrases: R: A+G, Y: C+T, M: A+C, K: G+T, S: G+C, W: A+T. DnaSP v 5.0 software was used for dividing the heterozygous loci into double sequence series (Librado and Rozas, 2009).

### Genetic Variation and Genetic Structure Analysis

The genetic diversity of the cpDNA and ITS sequences were analyzed in all four *Notopterygium* species using PERMUT v 1.0 software, where we calculated the genetic diversity within the population of each species (*h*_S_), total genetic diversity (*h*_T_), and population genetic differentiation coefficients *G*_ST_ and *N*_ST_ (Grivet and Petit, 2002).

In addition, ARLEQUIN v 3.5 (Excoffier and Lischer, 2010) software was used to perform analysis of molecular variance (AMOVA) for the cpDNA and ITS sequences. AMOVA partitioned the genetic differentiation among the populations *F*_ST_, within a population *F*_SC_, and among species *F*_CT_.

### Phylogenetic Analysis

Phylogenetic analyses of the cpDNA and ITS sequences were performed with MEGA v 7.0. JModeltest v 3.06 (Posada and Crandall, 1998) was used to filter the best evolutionary model (GTR+G). One-thousand bootstrap replicates were performed for the maximum likelihood (ML) and maximum parsimony (MP) models to obtain the phylogenetic tree. MrBayes v 3.2.3 was also used to conduct phylogenetic analyses of the cpDNA and ITS sequences based on the Bayesian criterion (Huelsenbeck and Ronquist, 2001). We set the random tree rotation as 10,000,000 generation, where each 1000 generations were kept to construct a phylogenetic tree, with a burn-in of 2500.

NETWORK v 5.0.0 (Polzin and Daneshmand, 2003) was used to construct median-joining networks of the cpDNA and ITS sequences. ArcGIS v 10.2 (Bader, 2005) was employed to draw the haplotype distribution map. BEAST v 1.7.5 (Drummond and Rambaut, 2007) was used to estimate the divergence times of the cpDNA haplotypes where we used the cpDNA evolutionary rates (1.0–3.0 × 10^-9^ s/s/y) recorded for other angiosperms to calibrate our datasets due to the lack of fossil evidence for *Notopterygium* plants (Wolfe et al., 1987). We employed the loose molecular clock method with an uncorrected log-normal distribution for the branch lengths. After a burn-in of 5,000,000 steps, all of the parameters were collected once every 1000 steps up to 50,000,000 Markov chain Monte Carlo (MCMC) algorithm steps. The convergence of the MCMC results was verified by using the Tracer v 1.5 program to check that the chain was balanced, where we then used the Tree Annotator v 1.7.5 program to obtain the best tree merging and Figtree v 1.3.1 (Rambaut, 2009) was employed to view the resulting tree.

### Population Dynamics Analysis

DnaSP v 5.0 was used to analyze the genetic diversity parameters, including the haplotype diversity (*H*_d_) (Nei and Tajima, 1981), nucleotide diversity (*π*) (Nei and Li, 1979), and number of haplotypes (*H*). We also used DnaSP v 5.0 to detect the mismatched distributions (Schneider and Excoffier, 1999) of cpDNA sequences in the four *Notopterygium* species. Population demographic expansions were tested using Arlequin v 3.5 (Excoffier and Lischer, 2010) and Tajima’s D (Tajima, 1989), Fu’s *F*_S_ (Fu, 1997), and Fu and Li’s *F^∗^* (Fu and Li, 1993) tests. We used the sum of the squared deviations between the observed and expected mismatches as well as Harpending’s raggedness index values (Rag) (Harpending, 1994) to determine the validity and significance level of the expansion model. According to the formula: *τ* =2*ut* (*τ* is the mismatch equilibrium expansion variable) (Rogers and Harpending, 1992), we calculated the expansion time *t*, where *u* is the mutation rate per generation calculated using the formula *u* = 2*μ*kg, where *μ* is the mutation rate per nucleotide per year, k is the total length of a cpDNA sequence, and g is the generation time. According to our field investigations, the generation time for *Notopterygium* species was 3 years.

In order to further determine the signs of demographic growth in the four *Notopterygium* species, we used LAMARC v 2.1.8 (Kuhner, 2006) to calculate the population growth parameter *g*. The MCMC algorithm was run for 100,000 generations and sampled every 200,000 steps, where the first 25% of the sampled trees were discarded as the burn-in.

### Species Distribution Modeling

We used MaxEnt v 3.3.3k (Phillips et al., 2006; Phillips and Dudík, 2008) to predict the current, last glacial maximum (LGM), last interglacial (LIG), and future distributions of two widespread *Notopterygium* species: *N. incisum* (148 distribution sites) and *N. franchetii* (80 distribution sites). The distribution sites of *Notopterygium* species were collected from previous studies as well as websites containing climate data and plant distributions. We also obtained some distribution sites based on field investigations. Bio-climatic environment data were downloaded from the WorldClim website^1^ at a resolution of 2.5 arc-minutes. Six bioclimatic environmental variables (Supplementary Table S7) with significant effects on *N. incisum* and *N. franchetii* were used to detect changes in the distribution ranges of plants. We set the number of replicates to 10 and the maximum number of iterations to 500 for MaxEnt modeling. The accuracy of the model’s performance was assessed based on the area under the receiver operating characteristic curve (AUC) (Fawcett, 2006).

## Results

### cpDNA Variations and Haplotype Distributions

Three chloroplast fragments (*matK*, *rbcL*, and *trn*S*-trn*G) were used to analyze 559 individuals from 74 populations of the four *Notopterygium* species. The total length of the fragments was 1605 bp, and the lengths of the *matK*, *rbcL*, and *trn*S*-trn*G regions were 669, 668, and 268 bp, respectively, which included 21, seven, and eight nucleotide mutation sites (Supplementary Table S3). The cpDNA regions were uniparental inherited markers so we combined the three chloroplast fragments in the subsequent population genetics analysis.

**FIGURE 2:**
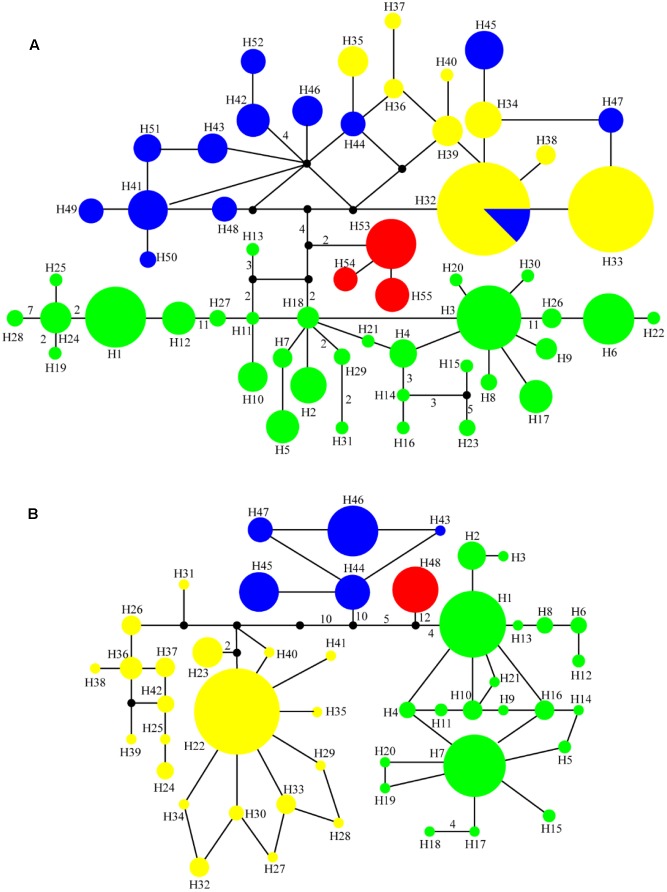
Median-joining networks for **(A)** 55 cpDNA haplotypes and **(B)** 48 ITS haplotypes in the genus *Notopterygium*. Each color denotes the four species in *Notopterygium* Boissieu, where green indicates *N. incisum*, yellow indicates *N. franchetii*, blue indicates *N. oviforme*, and red indicates *N. forrestii*. The numbers on the branches indicate the number of steps separating adjacent haplotypes.

In total, 55 cpDNA haplotypes were detected in the four *Notopterygium* species (**Figures 1**, **2**). Most of the haplotypes were species-specific, except the H32 haplotype was shared by *N. franchetii* and *N. oviforme. N. incisum* contained 31 haplotypes, where haplotypes H1–H7, H12, H18, and H26 were shared among populations, and the remainder were unique to each population. Populations from the southeast part of the QTP (G, J, Q, U, Z, HB, and HC; see **Table 1** for the site codes) had the highest haplotype diversity. *N. franchetii* had nine haplotypes with nine mutation sites. Populations from the west part of China (KG, KO, KZ, and YF) also had the highest haplotype diversity. *N. oviforme* had 12 haplotypes with 16 mutation sites, where the LE population had the highest haplotype diversity for this species. *N. forrestii* had three haplotypes with two mutation sites, where H55 was unique to the LCB population, and haplotypes H53 and H54 were shared by the other populations.

*Notopterygium oviforme* had the highest levels of genetic diversity and nucleotide diversity (*H*_d_ = 0.81, *π* = 0.0013), followed by *N. incisum* (*H*_d_ = 0.75, *π* = 0.00086) and *N. forrestii* (*H*_d_ = 0.39, *π* = 0.0002), whereas *N. franchetii* had the lowest level of diversity (*H*_d_ = 0.29, *π* = 0.00031) (**Table 2**).

**Table 2:** Gene diversity, nucleotide diversity, and haplotype frequencies of the ITS and cpDNA sequences for the four *Notopterygium* species.

Population code	cpDNA				ITS			
		
	Number of samples	*H*_d_ (*SD*)	*π* (*SD*) × 100	cpDNA Chlorotypes	Number of samples	*H*_d_ (*SD*)	*π* (*SD*) × 100	ITS types
A	13	0	0	H1(13)	13	0	0	H1(13)
B	5	0	0	H1(5)	5	0	0	H1(5)
C	10	0	0	H1(10)	10	0	0	H1(10)
D	10	0.36 (0.16)	0.02 (0.01)	H2(8)H3(2)	10	0	0	H2(10)
E	5	0	0	H3(5)	5	0.90 (0.16)	0.37 (0.07)	H1(1) H3(1) H4(1) H5(2)
F	10	0	0	H3(10)	10	0	0	H1(10)
G	12	0.53 (0.08)	0.07 (0.01)	H4(5) H5(7)	12	0.62 (0.12)	0.34 (0.05)	H6(2) H7(7) H8(3)
H	10	0	0	H6(10)	10	0.73 (0.12)	0.18 (0.04)	H4(2) H9(1) H10(5) H11(2)
I	10	0	0	H1(10)	10	0	0	H1(10)
J	10	0.80 (0.09)	0.07 (0.01)	H3(2) H5(4) H7(2) H8(2)	10	0.89 (0.08)	0.35 (0.04)	H4(1) H6(2) H7(3) H8(1) H12(2) H13(1)
L	10	0.20 (0.15)	0.01 (0.01)	H3(9) H7(1)	10	0	0	H7(10)
M	10	0	0	H9(4) H10(6)	10	0	0	H1(10)
N	10	0	0	H11(1) H12(9)	10	0	0	H7(10)
Q	10	0.68 (0.16)	0.23 (0.06)	H3(5) H12(1) H13(1) H14(1) H15(1) H16(1)	10	0.20 (0.15)	0.07 (0.05)	H7(9) H14(1)
S	10	0	0	H17(10)	10	0	0	H7(10)
T	5	0	0	H3(5)	5	0	0	H1(5)
U	5	0.80 (0.16)	0.06 (0.02)	H6(2) H18(2) H19(1)	6	0.87 (0.13)	0.24 (0.06)	H1(1) H7(2) H15(2) H16(1)
V	5	0	0	H6(5)	6	0.33 (0.22)	0.23 (0.15)	H7(4) H17(1) H18(1)
W	5	0	0	H3(1) H4(3) H18(1)	6	0.73 (0.16)	0.19 (0.04)	H1(3) H7(2) H16(1)
X	5	0.40 (0.24)	0.03 (0.02)	H3(3) H18(1) H20(1)	8	0.79 (0.15)	0.24 (0.08)	H7(4) H16(1) H19(1) H20(1) H21(1)
Y	2	0	0	H6(2)	2	0	0	H7(2)
Z	5	0.80 (0.14)	0.03 (0.07)	H3(1) H21(1) H22(1) H23(2)	5	0	0	H1(5)
HA	5	0.40 (0.24)	0.03 (0.02)	H24(4) H25(1)	5	0	0	H1(5)
HB	5	0.60 (0.18)	0.04 (0.01)	H26(1) H27(2) H28(2)	5	0.70 (0.22)	0.17 (0.06)	H1(3) H7(1) H16(1)
HC	5	0.70 (0.22)	0.01 (0.03)	H26(1) H29(2) H30(1) H31(1)	5	0.70 (0.22)	0.17 (0.06)	H1(1) H7(3) H16(1)
HF	5	0	0	H2(5)	5	0	0	H2(5)
HH	6	0.33 (0.22)	0.02 (0.01)	H6(5) H26(1)	6	0.33 (0.22)	0.06 (0.04)	H7(5) H16(1)
HI	5	0	0	H24(5)	5	0	0	H1(5)
*N. incisum*	208	0.75 (0.02)	0.086 (0.008)	-	217	0.71 (0.02)	0.25 (0.013)	-
KA	9	0	0	H32(9)	9	0	0	H22(9)
KC	10	0	0	H32(8) H33(2)	10	0	0	H22(10)
KD	10	0	0	H33(10)	10	0	0	H22(10)
KE	2	0	0	H34(2)	2	0	0	H23(2)
KF	7	0	0	H33(7)	7	0	0	H22(7)
KG	10	0.38 (0.18)	0.06 (0.03)	H32(1) H35(8) H36(1)	10	0.64 (0.10)	0.28 (0.04)	H24(4) H25(1) H26(5)
KH	10	0	0	H33(10)	10	0	0	H22(10)
KI	9	0	0	H32(9)	9	0.58 (0.18)	0.17 (0.06)	H22(6) H27(1) H28(1) H29(1)
KJ	10	0	0	H32(10)	10	0.78 (0.09)	0.31 (0.10)	H22(4) H30(2) H31(1) H32(3)
KK	10	0	0	H32(10)	10	0.64 (0.15)	0.23 (0.06)	H22(1) H30(1) H32(2) H33(6)
KL	10	0	0	H33(10)	10	0	0	H22(10)
KM	10	0	0	H34(10)	10	0	0	H23(10)
KN	2	0	0	H34(2)	2	0	0	H23(2)
KO	5	0.60 (0.18)	0.04 (0.01)	H32(3) H36(2)	5	0	0	H22(5)
KP	5	0	0	H32(5)	5	0.70 (0.22)	0.18 (0.06)	H22(3) H32(1) H34(1)
KQ	10	0	0	H32(8) H33(2)	10	0	0	H22(10)
KR	2	0	0	H37(2)	2	0	0	H22(2)
KS	5	0	0	H33(5)	5	0	0	H22(5)
KV	6	0	0	H33(6)	6	0	0	H22(6)
KX	6	0	0	H33(6)	6	0	0	H22(6)
KZ	6	0.60 (0.13)	0.04 (0.01)	H33(3) H38(3)	6	0.33 (0.22)	0.06 (0.04)	H22(5) H35(1)
YA	5	0	0	H32(5)	5	0	0	H22(5)
YB	5	0	0	H33(5)	5	0	0	H22(5)
YC	5	0	0	H39(5)	9	0.64 (0.13)	0.12 (0.03)	H36(5) H37(3) H38(1)
YD	5	0	0	H32(5)	5	0.70 (0.22)	0.20 (0.08)	H36(3) H37(1) H39(1)
YE	5	0	0	H32(4) H33(1)	6	0.60 (0.22)	0.11 (0.05)	H22(4) H40(1) H41(1)
YF	5	0.40 (0.24)	0.03 (0.02)	H39(4) H40(1)	6	0.60 (0.22)	0.15 (0.06)	H36(1) H37(1) H42(4)
YK	5	0	0	H33(5)	5	0	0	H22(5)
YM	5	0	0	H32(1) H33(4)	5	0	0	H22(5)
*N. franchetii*	194	0.29 (0.04)	0.031 (0.006)	-	200	0.55 (0.042)	0.364 (0.037)	-
LA	10	0	0	H41(10)	10	0.20 (0.15)	0.03 (0.03)	H43(1) H44(9)
LB	10	0	0	H42(10)	10	0	0	H45(10)
LC	10	0.47(0.13)	0.03 (0.01)	H43(7) H44(3)	10	0.20 (0.15)	0.03 (0.03)	H44(1) H45(9)
LD	17	0	0	H45(17)	17	0	0	H46(17)
LE	15	0.56 (0.10)	0.12 (0.02)	H41(1) H46(9) H47(5)	15	0	0	H46(15)
LF	10	0	0	H32(10)	10	0	0	H47(10)
LG	2	0	0	H44(2)	2	0	0	H44(2)
LK	5	0	0	H48(5)	5	0	0	H46(5)
LO	5	0	0	H49(5)	5	0	0	H46(5)
LP	5	0.60 (0.18)	0.08 (0.02)	H50(2) H51(3)	5	0	0	H44(5)
LQ	5	0	0	H41(5)	5	0	0	H46(5)
LT	5	0.40 (0.24)	0.03 (0.02)	H42(1) H51(4)	5	0.40 (0.24)	0.07 (0.04)	H44(1) H45(4)
LU	5	0	0	H52(5)	6	0.33 (0.22)	0.06 (0.04)	H44(1) H45(5)
*N. oviforme*	104	0.81 (0.03)	0.13 (0.01)	-	105	0.69 (0.03)	0.24 (0.007)	-
LCA	10	0	0	H53(7) H54(3)	10	0	0	H48(10)
LCB	10	0	0	H55(10)	10	0	0	H48(10)
LCC	10	0	0	H53(10)	10	0	0	H48(10)
LCD	10	0	0	H53(9) H54(1)	10	0	0	H48(10)
*N. forrestii*	40	0.39 (0.07)	0.02 (0.00)	-	40	0	0	-
Total	546	0.85 (0.01)	0.368 (0.005)	-	559	0.885 (0.007)	2.81 (0.035)	-


*N, number of samples; *H*_*d*_, gene diversity; *π*, nucleotide diversity averaged across loci.*

### ITS Sequence Variation

The total length of the sequenced ITS region was 593 bp and 48 haplotypes were identified with 66 nucleotide mutation sites (**Figure 2** and Supplementary Table S4). All of the ITS haplotypes were species-specific in the four *Notopterygium* species. The total haplotype diversity (*H*_d_) and *π* values for *N. incisum, N. franchetii*, and *N. oviforme* were 0.71 and 0.0025, 0.55 and 0.00364, and 0.69 and 0.0024, respectively. *N. incisum* populations from the southeast part of the QTP (E, G, H, J, U, W, X, HB and HC) had the highest haplotype diversity in this species, and haplotypes H1 and H7 had the highest distribution frequencies. *N. franchetii* populations from KG, KI, KJ, KK, KP, YC, YD, YE, and YF had the highest haplotype diversity in this species, and haplotype H22 had the highest frequency. In addition, *N. oviforme* and *N. forrestii* exhibited low haplotype diversity in terms of their ITS sequences (**Table 2** and Supplementary Figure S1).

### Genetic Diversity and Structure

The total genetic diversity (*h*_T_) values based on the cpDNA datasets for *N. incisum*, *N. franchetii*, *N. oviforme*, and *N. forrestii* were 0.939, 0.766, 0.961, and 0.623, respectively, where *N. incisum* had the highest levels for *h*_S_ (0.404) and *h*_T_, whereas *N. forrestii* had the lowest level of diversity (*h*_S_ = 0.167; *h*_T_ = 0.623) (**Table 3**). In addition, we calculated the genetic differentiation coefficients *G*_ST_ and *N*_ST_ for the four species. The U statistic (Gaussian test 1000 times) showed that *N*_ST_ was significantly larger than *G*_ST_ for *N. incisum* and *N. oviforme* (*P* < 0.05), thereby indicating that these two species exhibited significant phylogeographic structuring (**Table 3**).

**Table 3:** Genetic diversity and differentiation analyses for cpDNA and ITS variations in *Notopterygium* species.

Species	cpDNA				ITS			
		
	*h*_S_(SE)	*h*_T_(SE)	*G*_ST_(SE)	*N*_ST_(SE)	*h*_S_(SE)	*h*_T_(SE)	*G*_ST_(SE)	*N*_ST_(SE)
*N. incisum*	0.404	0.939	0.569	0.703^∗∗^	0.267	0.725	0.632	0.516
	0.073	0.022	0.073	0.069	0.065	0.05	0.078	0.055
*N. franchetii*	0.203	0.766	0.735	0.69	0.154	0.557	0.723	0.788
	0.05	0.045	0.059	0.11	0.05	0.099	0.077	0.05
*N. oviforme*	0.242	0.961	0.748	0.975^∗∗^	0.05	0.693	0.928	0.965^∗^
	0.078	0.029	0.078	0.017	0.029	0.081	0.039	0.019
*N. forrestii*	0.167	0.623	0.733	0.718	-	-	-	-
	0.111	0.177	0.244	0.262	-	-	-	-


**h*_*S*_, estimates of average genetic diversity within populations; *h*_*T*_, total genetic diversity; *G*_*ST*_ and *N*_*ST*_, inter-population differentiation; (SE), mean ± SE in parentheses; ^∗^*P* < 0.05, ^∗∗^*P* < 0.01 (both indicate that *N*_*ST*_ differs significantly from *G*_*ST*_); -, no data.*

AMOVA analysis of the cpDNA datasets detected genetic variations among the four species (*F*_CT_ = 0.5804) (Supplementary Table S5). In the four individual species, the genetic variations among populations (*N. incisum, F*_ST_ = 0.8196; *N. franchetii*, *F*_ST_ = 0.8391; *N. oviforme*, *F*_ST_ = 0.8474; and *N. forrestii*, *F*_ST_ = 0.7585) were significantly higher than those within populations (Supplementary Table S5). In addition, the AMOVA results obtained for the ITS sequences indicated similar genetic differentiation patterns to those based on the cpDNAs, where the differences among species in terms of the variation in the ITS were as high as 92% (*F*_CT_ = 0.9287) (Supplementary Table S6).

### Phylogenetic Relationships

**FIGURE 3:**
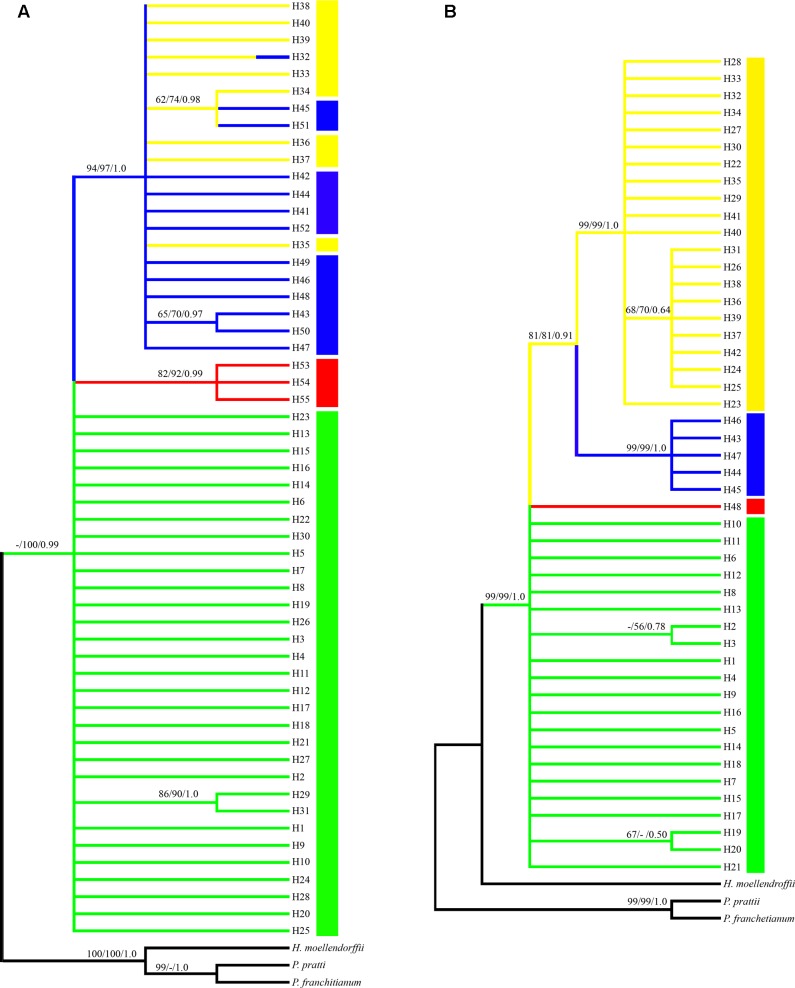
Phylogenetic tree for the **(A)** 55 cpDNA haplotypes and **(B)** 48 ITS haplotypes. Each color denotes the four species in the genus *Notopterygium* Boissieu, where green indicates *N. incisum*, yellow indicates *N. franchetii*, blue indicates *N. oviforme*, and red indicates *N. forrestii*. Posterior probabilities are shown above the branches and bootstrap support below the branches (when > 50% for each case).

Phylogenetic trees of the cpDNA haplotypes were constructed based on the ML, MP, and Bayesian inference methods, which showed that the topological structures obtained were basically the same using the three methods (**Figure 3**). The four species of *Notopterygium* formed a larger monophyletic clade with high bootstrap support, where *N. franchetii* and *N. oviforme* were sisters. The median-joining network diagram produced using the cpDNA datasets was consistent with the phylogenetic analysis (**Figure 2**). The major haplotypes with the highest distribution frequencies (H1, H11, H32, H33, H41, and H53) were located in the central positions of the network. However, the phylogenetic relationships of the ITS sequences differed from those of the cpDNA sequences. No haplotypes were shared among the four *Notopterygium* species and each species formed its own individual branch (**Figure 3**) in the ITS tree. In addition, in order to confirm the phylogenetic positions of all four species considered in this study, we analyzed the other two species in the genus *Notopterygium*, i.e., *N. tenuifolium* and *N. pinnatiinvolucellatum*. Phylogenetic analyses based on variations in the chloroplast *rbcL* sequence showed that the four species considered in this study, i.e., *N. incisum*, *N. franchetii*, *N. oviforme*, and *N. forrestii*, were more closely related than *N. tenuifolium* and *N. pinnatiinvolucellatum* (Supplementary Figure S2).

### Population Dynamics History and Divergence Time

Based on the cpDNA sequences, we performed various mathematical analyses to determine the population histories of the four *Notopterygium* species (**Table 4** and Supplementary Figure S3). The mismatch distribution model had a single peak, with negative Tajima’s *D* and Fu’s *F*_S_ values for *N. incisum* and *N. franchetii*, which suggested that these two species had experienced rapid range expansions. The larger population growth indexes for *N. incisum* (*g* = 809) and *N. franchetii* (*g* = 2810.736) were also consistent with rapid population expansions. By contrast, *N. oviforme* and *N. forrestii* had bimodal mismatch distributions with positive Tajima’s *D* and Fu’s *F*_S_ values, where these results indicated that they did not experience expansion events. Therefore, we estimated the expansion times for *N. incisum* and *N. franchetii* as about 128–43 Kya and 51–17 Kya in the Pleistocene, respectively (**Table 5**).

**Table 4:** Results of cpDNA mismatch distribution and neutrality tests for the four *Notopterygium* species.

Species	Mismatch distribution				Neutrality tests			
		
	θ_0_	θ_1_	SSD (*P*-value)	Rag (*P*-value)	G	Tajima’s *D*	Fu and Li’s F^∗^	Fu’s *F*_S_
*N. incisum*	3.6	12.48047	0.05068 (0.08)	0.04751 (0.03)	809	-1.46084	-0.26004	-9.305
*N. franchetii*	0.000	99999	0.01084 (0.014)	0.10871 (0.001)	2810.736	-1.27738	-0.38051	-3.278
*N. oviforme*	0.0	11.04492	0.02277 (0.09)	0.06534 (0.08)	614.1456	0.52628	1.23674	0.081
*N. forrestii*	0.0	99999	0.01913 (0.05)	0.17541 (0.11)	562.4275	0.90802	0.76302	1.292


*θ_*0*_ and θ_*1*_ are the pre-expansion and post-expansion populations sizes, respectively; SSD, sum of squared deviations; Rag, Harpending’s Raggedness index; G, population size index, were G = -10 indicates that the population size might be shrinking and G = 200 indicates that the population size might be growing rapidly; Tajima’s *D*, Fu and Li’s *D*^∗^, Fu and Li’s F^∗^, and Fu’s *F_*S*_* were significant at *P* < 0.05.*

**Table 5:** Ages (years ago) of putative expansion events estimated by mismatch analyses.

Species	T (Mya)	*t* (μ = 1 × 10^-9^)	*t* (μ = 3 × 10^-9^)
*N. incisum*	2.46 (0.88–6.03) 1.0	127725.9 (45690.5–313084.1)	42575.29 (15230.18–104361.4)
*N. franchetii*	(0.79688–1.33594)	51921.08 (41374.87–69363.45)	17307.03 (13791.62–23121.15)


*τ, time in number of generations elapsed since the sudden expansion episode; t, absolute time in years.*

**FIGURE 4:**
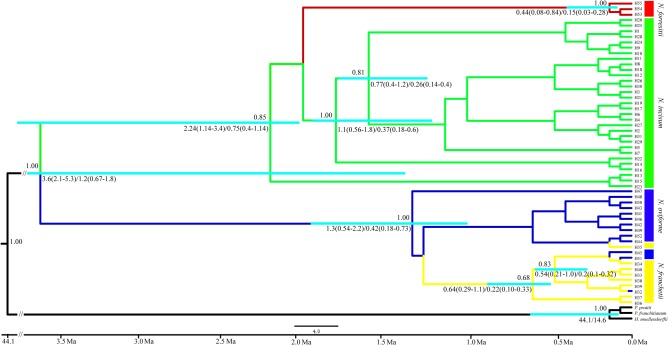
Chronogram for the four *Notopterygium* species obtained using BEAST based on the plastid sequences. The turquoise color bar indicates the 95% highest posterior density (HPD) credibility intervals for node ages (million years ago, Mya). Posterior probabilities are labeled above the line, and the mean divergence dates and 95% HPDs are labeled below the line.

We estimated the divergence times between the four species of *Notopterygium* based on a range of mutation rates (1.0–3.0 × 10^-9^ s/s/y). The first divergence among the four species occurred between approximately 3.6 Mya (95% highest posterior density (HPD), 2.1–5.3 Mya) and 1.2 Mya (95% HPD, 0.67–1.8 Mya), whereas the estimated divergence between *N. forrestii* and *N. incisum* occurred between 2.24 Mya (95% HPD, 1.14–3.4 Mya) and 0.75 Mya (95% HPD, 0.4–1.14 Mya). In addition, the divergence between the major lineages of *N. franchetii* and *N. oviforme* occurred between 1.3 Mya (95% HPD, 0.54–2.2 Mya) and 0.42 Mya (95% HPD, 0.18–0.73 Mya) (**Figure 4**).

### Species Distribution Modeling

**FIGURE 5:**
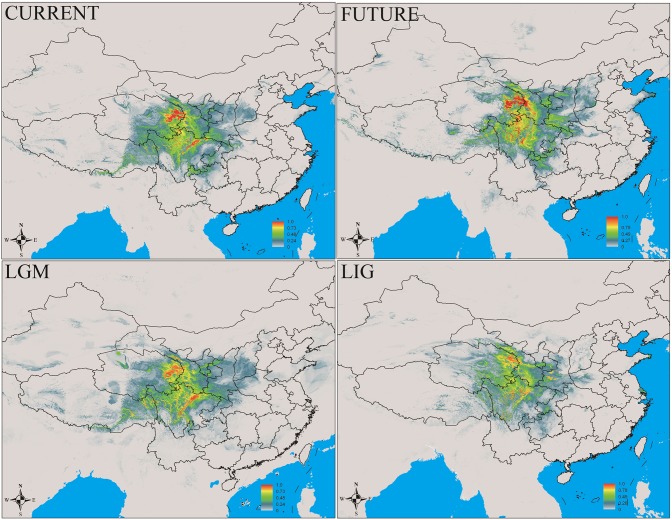
Geographic distribution pattern obtained for *N. incisum* using MaxEnt. LIG, last interglacial period; LGM, last glacial maximum.

**FIGURE 6:**
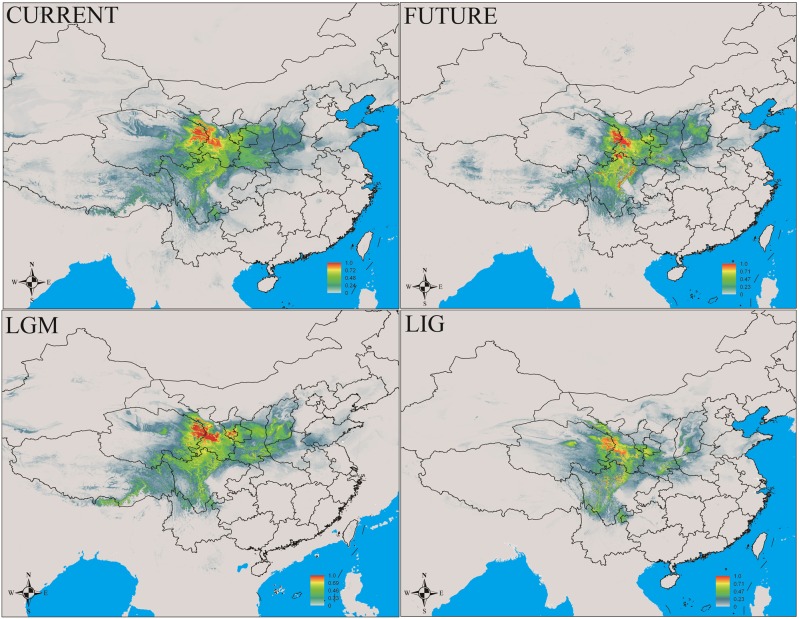
Geographic distribution pattern obtained for *N. franchetii* using MaxEnt. LIG, last interglacial period; LGM, last glacial maximum.

In this study, MaxEnt modeling had the highest predictive capacity (AUC > 0.9) for the two widely distributed *Notopterygium* species (*N. franchetii* and *N. incisum*). The distribution ranges predicted for these two species were consistent with the current geographic distributions in the QTP and adjacent areas (**Figures 5**, **6** and Supplementary Table S7). Species distribution modeling also showed that the range of *N. incisum* was limited in the LIG period whereas it expanded very rapidly in the LGM period. However, there were no significant changes in the distribution range from the LGM until the current period for this species. For *N. franchetii*, MaxEnt modeling suggested that the distribution range of this species increased very rapidly from the LIG until the LGM period. However, it was interesting that the distribution range of *N. franchetii* did not change greatly from the LGM until the current period.

## Discussion

### Genetic Diversity and Structure

In the current study, our analysis of the cpDNA sequences showed that *N. oviforme* had the highest level of genetic diversity (*H*_d_ = 0.81, *π* = 0.0013), followed by *N. incisum* (*H*_d_ = 0.75, *π* = 0.00086) and *N. forrestii* (*H*_d_ = 0.39, *π* = 0.0002), whereas *N. franchetii* had the lowest level of diversity (*H*_d_ = 0.29, *π* = 0.00031) (**Table 2**). However, the results were different according to the ITS sequence analysis, where *N. incisum* had the highest diversity (*H*_d_ = 0.71, *π* = 0.0025), followed by *N. oviforme* (*H*_d_ = 0.69, *π* = 0.0024) and *N. franchetii* (*H*_d_ = 0.55, *π* = 0.0036), whereas *N. forrestii* exhibited no variation (**Table 2**). In addition, all four *Notopterygium* species had high levels of genetic differentiation, where the genetic variations in the cpDNA and ITS sequences mainly occurred among the populations within each species (**Table 3** and Supplementary Tables S5, S6).

In general, *N. franchetii* has the most extensive natural geographic distribution range, but we found that its cpDNA and ITS sequences had low diversity. We consider that this low diversity may be due to harvesting and climatic changes, where many of the natural populations of *N. franchetii* have become extinct because of habitat destruction, thereby causing low diversity and high genetic differentiation (Lowe et al., 2005). *N. incisum* is another widely distributed species but we found that it had a high level of genetic diversity compared with other three species, which may be explained by the less extensive destruction of the wild populations of this species. According to the field investigations, we found that this species generally occurs in higher altitude areas (≥3000 m) compared with other *Notopterygium* species, and thus its less frequent harvesting might explain the high genetic variation (Wang G.N. et al., 2014). In addition, the high level of genetic diversity in *N. oviforme* according to this study might be explained by the lower altitude range of this species (1700–3200 m), which is consistent with a previous report of high species diversity at low altitudes (Lu et al., 2012). The lower genetic diversity of *N. forrestii* may be due to its narrow geographical distribution, where the smaller localized populations can interbreed and the gene flow is greater, thereby leading to a low level of diversity.

These *Notopterygium* species may also have been affected by adverse environmental changes in the high altitude QTP and adjacent areas. Thus, repeated climatic oscillations and geological events may have led to genetic drift and the fragmentation of habitats, thereby reducing their diversity (*N. oviforme* had slightly higher diversity compared with the other three species) and causing a high level of genetic differentiation among the populations of *Notopterygium* species (Hamrick and Loveless, 1989).

### Relationships among Species

Phylogenetic analysis based on the cpDNA and ITS haplotypes showed that all four *Notopterygium* species formed a monophyletic clade with high bootstrap support (**Figure 3** and Supplementary Figure S2). *N. franchetii* and *N. oviforme* shared a common branch in the phylogenetic tree based on the cpDNA sequences, and they also shared cpDNA haplotype H32. However, there were no shared ITS haplotypes among the four *Notopterygium* species, where each species formed an individual clade in the ITS tree. Thus, the ITS marker could identify the species at greater resolution than the cpDNA fragments. In general, cpDNA is a uniparentally inherited region whereas nuclear ITS fragments are biparentally inherited markers in most angiosperms, so ITS markers are superior for discriminating lineages and species than cpDNA fragments (Fan et al., 2011; Wang et al., 2012).

In addition, the shared cpDNA H32 haplotype was found in the parapatric populations (LF and YA) of *N. franchetii* and *N. oviforme*. These parapatric geographic distributions may have provided the opportunity for interspecific gene flow and hybridization among the two species. According to the field observations, we found that these two species have overlapping flowering times, which may have facilitated genetic introgression among these species. Previous studies have also suggested the occurrence of hybridization among species distributed in the same geographic regions and subsequent backcrosses with one of the parental species, where these processes resulted in high levels of shared plastid genotypes (Hamzeh et al., 2006; Li et al., 2013; Wang Z. et al., 2014). However, it is also possible that incomplete lineage sorting could have lead to the sharing of cpDNA haplotypes among species. The perennial herb *Notopterygium* species have large population sizes and long generation times, which may have led to the sharing of ancestral polymorphisms among species.

### Species Divergence and Population Dynamics History

Mountain barriers may play a key role in speciation and diversification because their topographic complexity can lead to ecological stratification and environmental heterogeneity (Fjeldså et al., 2012). In the present study, we estimated the divergence time of the four *Notopterygium* species based on three cpDNA fragments, which showed that their divergence occurred between about 3.6 Mya (95% HPD, 2.1–5.3 Mya) and 1.2 Mya (95% HPD, 0.67–1.8 Mya) in the Pliocene and Pleistocene periods. The divergence of *N. forrestii* and *N. incisum* was estimated as occurring between 2.24 Mya (95% HPD, 1.14–3.4 Mya) and 0.75 Mya (95% HPD, 0.4–1.14 Mya) in the early to middle Pleistocene period (**Figure 4**). We suggest that the divergence of the four *Notopterygium* species was significantly related to the uplift of the QTP. Previous studies and geological data indicated that the uplift of the QTP started in the Oligocene to Miocene (25–17 Mya), middle of the Miocene (15–13 Mya), late Miocene (8–7 Mya), or in the Pliocene to early Pleistocene period (3.6–1.8 Mya) (Harrison et al., 1992; Coleman and Hodges, 1995; Shi et al., 1998; Spicer et al., 2003). During the uplift of the QTP and adjacent Himalayan mountains, long-term geological events generated great environmental differences, which might have triggered the diversification of species in the genus *Notopterygium*. Other studies have also shown that the recent extensive uplift of the QTP and adjacent mountains triggered the lineage divergence and evolution of many herb species due to geographical isolation and climatic changes (Lei et al., 2007, 2015).

In addition, dramatic variations in the environment and climate might have affected the genetic structure and geographic distributions of the *Notopterygium* species. MaxEnt modeling showed that the two cold-tolerant species comprising *N. incisum* and *N. franchetii* exhibited significant range expansions from the LIG to the LGM period (**Figures 5**, **6**). The mismatch analysis, neutrality test, and population growth index results also supported similar expansions by these two species. Therefore, we estimated the expansion times for *N. incisum* and *N. franchetii* as about 128–43 and 51–17 Kya, respectively, during the late Pleistocene (**Table 5**). We showed that the geographic ranges of these two species increased significantly during the ice ages in the Pleistocene. Demographic expansions of cold-tolerant tree species during the glacial periods have also been reported in high altitude areas of the QTP (Qiu et al., 2011; Wen et al., 2014). Moreover, repeated founder and bottleneck effects during the expansion processes may explain the low genetic variation in the two species. By contrast, we found that the populations of *N. incisum* and *N. franchetii* had high levels of genetic diversity in the southeast part of the QTP. For example, some *N. incisum* populations in Qinghai (population G), Sichuan (populations J, Q, U, and X), and Gansu (population Z) had high diversity and many more unique haplotypes. The *N. franchetii* populations in Sichuan (populations KG and YF) and Gansu (populations KO and KZ) also had high genetic diversity and a rich abundance of haplotypes (**Table 2**). In addition, the LE population of *N. oviforme* and the LCA population of *N. forrestii* had high levels of haplotype diversity. These areas may have provided important glacial refugia for these endemic perennial herb species. Mountain areas at low latitudes can also provide relatively stable environmental conditions according to the “ecological stability hypothesis” (Qu et al., 2014; Lei et al., 2015), which implies that these populations should have high genetic diversity and a rich diversity of haplotypes (Tzedakis et al., 2002; Abbott and Brochmann, 2003; Petit et al., 2003; Schonswetter et al., 2005). Similar results have been obtained for other organisms, such as birds (Lei et al., 2007), mammals (Wu et al., 2005), spiders (Meng et al., 2008), and aphids (Huang et al., 2010).

### Conservation Strategies for Endangered *Notopterygium* Species

The genus *Notopterygium* comprises unique perennial herbaceous plants with medicinal applications in China (Zhou et al., 2010). These species have high economic value so the market demand is great, especially for *N. incisum* and *N. franchetii*. However, in recent years, due to their continuous harvesting, slow growth rate, and low reproductive capacity, the natural populations of these *Notopterygium* species have been greatly depleted (Zhou et al., 2010). According to field investigations, we found that many of the previously recorded natural populations of species in the genus *Notopterygium* were extinct, and thus these important resources require urgent conservation and management.

According to the results of our population genetics analysis, we propose that the natural populations of wild *Notopterygium* species should be protected *in situ*, especially in the natural refugia areas (i.e., populations J, Q, U, and X of *N. incisum*; populations KO and KZ of *N. franchetii*; population LE of *N. oviforme*; and population LCA of *N. forrestii*). In addition, it is necessary to control all activities that deplete the sizes of the populations (e.g., illegal harvesting) and genetic fragmentations (e.g., habitat loss) (Hamilton et al., 2012).

In order to conserve the populations of a species, it is necessary to understand the genetic diversity and population structure of the natural populations (Schmitt, 2007). In this study, we found that the genetic variability and haplotype diversity were low for the widely distributed species, thereby indicating that the habitats have been destroyed or fragmented for these species, where interbreeding has occurred with nearby populations of individuals, thereby reducing the haplotype diversity. It is also necessary to protect the populations in different regions in order to increase the genetic links among populations. Finally, the mature seeds from each population should be collected and artificially planted with other populations in order to improve the habitats and to strengthen the gene exchange among populations (Cabrera-Toledo et al., 2012).

## Author Contributions

Z-HL designed and conceived the study. KS and YJ performed the experiments. Z-HL, KS, YJ, F-LC, and UZ contributed materials/analysis tools. KS and Z-HL wrote the manuscript. KS, YJ, and Z-HL revised the manuscript. All of the authors finally approved the manuscript.

## Conflict of Interest Statement

The authors declare that the research was conducted in the absence of any commercial or financial relationships that could be construed as a potential conflict of interest.

## Footnotes

1 http://www.wordclim.org
