# Supplementary material for: Effects of Mountain Uplift and Climatic Oscillations on Phylogeography and Species Divergence in Four Endangered Notopterygium Herbs
Source: Front Plant Sci. 2017 Nov 8;8:1929. doi: 10.3389/fpls.2017.01929 (PMC5682393; doi:10.3389/fpls.2017.01929)
Supplement: Supplementary file 1 [file Table_1.DOC]

**Supporting Information**

**Table S1.** Details of the primer sequences and temperatures.

**Table S2.** PCR reaction temperatures and times.

**Table S3.** Variable sites in the aligned sequences of the three cpDNA fragments (*matK, rbcL*, and *trn*S*-trn*G).

**Table S4.** Variable sites in the aligned ITS sequences.

**Table S5.** Analysis of molecular variance (AMOVA) within *Notopterygium* species based on the cpDNA datasets.

**Table S6.** Analysis of molecular variance (AMOVA) within *Notopterygium* based on the ITS datasets.

**Table S7.** Ecological variables for *Notopterygium incisum* and *Notopterygium franchetii*.

**Figure S1.** Geographic distribution of ITS haplotypes in *Notopterygium* species. Each circle represents a population and each color represents a haplotype. The colored outlines of the circles distinguish the four species, where green indicates *Notopterygium incisum*, yellow indicates *Notopterygium franchetii*, blue indicates *Notopterygium oviforme*, and red indicates *Notopterygium forrestii*.

**Figure S2.** Phylogenetic relationships between cpDNA fragment *rbcL* for the six *Notopterygium* species inferred from maximum likelihood analyses. *Pleurospermum* *prattii* and *Pleurospermum franchetianum* were used as outgroups. Numbers above the lines on the left indicate the maximum likelihood bootstrap values for clade > 50%.

**Figure S3.** Mismatch distribution analyses for cpDNA datasets in *Notopterygium* species: (a) *Notopterygium incisum*, (b) *Notopterygium franchetii*, (c) *Notopterygium oviforme*, and (d) *Notopterygium forrestii*. The blue line indicates the observed distribution of pairwise differences among the samples and the green line indicates the distribution expected under the sudden expansion model.

**Table S1.** Detail of Primers and their sequences with temperatures.

| **Gene amplification region** | **Forward (F) and reverse (R)** | **Primers sequence (5′-3′)** | **Tm (°C)** |
| --- | --- | --- | --- |
| *trn*S-*trn*G | *trn*S-*trn*G_F | GCCGCTTTAGTCCACTCAGC | 61.9 |
|  | *trn*S-*trn*G_R | GAACGAATCACACTTTTACCAC | 56.4 |
| *matkK* | *matK*3Fkim | CGTACAGTACTTTTGTGTTTACGAG | 58.7 |
|  | *matK*1Rkim | ACCCAGTCCATCTGGAAATCTTGGTTC | 63.5 |
| *rbcL* | *rbcL*1F | ATGTCACCACAAACAGAAAC | 53.7 |
|  | *rbcL*724R | TCGCATGTACCTGCAGTAGC | 59.8 |
| ITS | ITS4 | TCCTCCGCTTATTGATATGC | 55.8 |
|  | ITS5 | GGAAGGAGAAGTCGTAACAAGG | 60.1 |
|  | ITSR | CCTGCGGAAGGATCATTGTC | 59.8 |
| *trnL*-*trnF* | *trnL*-*trnF_*F  *trnL*-*trnF_*R | GGTTCAAGTCCCTCTATCCC  ATTTGAACTGGTGACACGAG | 58.5  60.2 |
| *inf*A-*rpl*36 | *inf*A-*rpl*36_F | AAGGAAATCCAAAAGGAACTCG | 56.8 |
|  | *inf*A-*rpl*36_R | GGRTTGGAACAAATTACTATAATTCG | 58.8 |

**Table S2.** PCR reaction temperatures and times.

| **Procedure step** | **Temperature (°C)** | **Time** |
| --- | --- | --- |
| Pre-degradation | 94 | 5 min |
| Degradation | 94 | 40 s |
| Annealing | *trnS-trnG*: 58 | 40 s |
|  | *matK*: 60 |  |
|  | *rbcL*: 52 |  |
|  | ITS: 52 |  |
| Extension | 72 | 90 s |
| Cycles |  | 35 |

**Table S3.** Variable sites in the aligned sequences of the three cpDNA fragments (*matK, rbcL*, and *trn*S*-trn*G).

| **cp -DNA Hap- loty- pes** | ***matK*** | | | | | | | | | | | | | | | | | | | | |  | ***rbcL*** | | | | | | |  | ***trn*S *- trn*G** | | | | | | | | | | |
| --- | --- | --- | --- | --- | --- | --- | --- | --- | --- | --- | --- | --- | --- | --- | --- | --- | --- | --- | --- | --- | --- | --- | --- | --- | --- | --- | --- | --- | --- | --- | --- | --- | --- | --- | --- | --- | --- | --- | --- | --- | --- |
|  | 0 | 0 | 0 | 0 | 0 | 0 | 0 | 0 | 0 | 0 | 0 | 0 | 0 | 0 | 0 | 0 | 0 | 0 | 0 | 0 | 0 |  | 0 | 0 | 0 | 0 | 0 | 1 | 1 |  | 1 | 1 | 1 | 1 | 1 | 1 | 1 | 1 | 1 | 1 | 1 |
|  | 0 | 0 | 1 | 1 | 1 | 1 | 1 | 1 | 2 | 2 | 2 | 2 | 3 | 3 | 3 | 4 | 4 | 4 | 5 | 6 | 6 |  | 6 | 7 | 7 | 8 | 9 | 1 | 1 |  | 3 | 3 | 3 | 4 | 4 | 4 | 4 | 4 | 4 | 4 | 4 |
|  | 4 | 5 | 1 | 3 | 3 | 4 | 4 | 9 | 1 | 4 | 5 | 7 | 4 | 7 | 9 | 3 | 4 | 6 | 6 | 3 | 4 |  | 8 | 2 | 9 | 6 | 3 | 0 | 0 |  | 4 | 7 | 9 | 4 | 4 | 4 | 4 | 4 | 5 | 5 | 5 |
|  | 7 | 9 | 4 | 0 | 2 | 4 | 5 | 7 | 3 | 5 | 4 | 7 | 2 | 6 | 4 | 8 | 7 | 4 | 9 | 8 | 5 |  | 0 | 6 | 4 | 9 | 5 | 1 | 9 |  | 9 | 3 | 1 | 5 | 6 | 7 | 8 | 9 | 0 | 1 | 2 |
| H1 | G | G | C | C | T | T | C | G | T | C | T | C | A | T | C | G | T | T | T | T | C |  | C | A | C | G | G | T | G |  | T | G | A | - | - | - | - | - | - | - | - |
| H2 | . | . | . | . | . | . | . | A | . | . | . | . | . | . | . | T | . | . | . | . | . |  | . | . | . | . | . | . | . |  | . | . | . | T | G | A | A | A | A | A | A |
| H3 | . | . | . | . | . | . | . | . | . | . | . | . | . | . | . | . | . | . | . | . | . |  | . | . | . | . | . | . | . |  | . | . | . | . | . | . | . | . | . | . | . |
| H4 | . | . | . | . | . | . | . | . | . | . | . | . | . | . | . | . | . | . | . | . | . |  | . | . | . | . | . | . | . |  | . | . | . | . | . | . | . | . | . | . | . |
| H5 | . | . | . | . | . | . | . | . | . | . | . | . | . | C | . | . | . | . | . | . | . |  | . | . | . | . | . | . | . |  | C | . | . | . | . | . | . | . | . | . | . |
| H6 | . | . | . | . | . | . | . | . | . | . | . | . | . | . | T | . | . | . | . | . | . |  | . | . | . | . | . | . | . |  | . | . | . | . | . | . | . | . | . | . | . |
| H7 | . | . | . | . | . | . | . | . | . | . | . | . | . | . | . | . | . | . | . | . | . |  | . | . | . | . | . | . | . |  | C | . | . | . | . | . | . | . | . | . | . |
| H8 | . | . | . | . | . | . | . | . | . | . | . | . | . | C | . | . | . | . | . | . | . |  | . | . | . | . | . | . | . |  | . | . | . | . | . | . | . | . | . | . | . |
| H9 | . | . | . | . | . | . | . | . | . | . | . | . | . | . | . | G | . | . | . | . | . |  | . | . | . | . | . | . | . |  | . | . | . | . | . | . | . | . | . | . | . |
| H10 | . | . | . | . | . | . | . | . | . | . | . | . | . | . | . | G | . | . | . | . | . |  | . | . | . | . | . | . | . |  | . | . | . | . | . | . | . | . | . | . | . |
| H11 | . | . | . | . | . | . | . | . | . | . | . | . | . | . | . | . | . | . | . | . | . |  | . | . | . | . | . | . | . |  | . | . | . | . | . | . | . | . | . | . | . |
| H12 | . | . | . | . | . | . | . | . | . | . | . | . | . | . | . | . | . | . | . | . | . |  | . | . | . | . | . | . | . |  | . | . | . | - | - | - | - | - | - | - | - |
| H13 | . | . | . | G | . | . | T | . | . | . | . | . | G | . | . | . | A | . | . | . | . |  | . | . | . | . | . | . | . |  | . | . | . | . | . | . | . | . | . | . | . |
| H14 | . | . | . | . | . | . | . | . | . | . | . | . | . | . | . | . | . | . | . | . | . |  | . | . | . | . | . | . | . |  | . | . | . | . | . | . | . | . | . | . | . |
| H15 | . | . | . | G | . | . | T | . | . | . | . | . | G | . | . | . | A | . | . | . | . |  | . | . | . | . | . | . | . |  | . | . | . | . | . | . | . | . | . | . | . |
| H16 | . | . | . | . | . | . | . | . | . | . | . | . | . | . | . | . | . | . | . | . | . |  | . | . | . | . | . | . | . |  | . | . | . | . | . | . | . | . | . | . | . |
| H17 | . | . | . | . | . | . | . | . | . | . | . | . | . | . | . | . | . | . | . | . | . |  | . | . | . | . | . | . | . |  | . | . | . | G | . | . | . | . | . | . | . |
| H18 | . | . | . | . | . | . | . | . | . | . | . | . | . | . | . | . | . | . | . | . | . |  | . | . | . | . | . | . | . |  | . | . | . | . | . | . | . | . | . | . | . |
| H19 | . | . | . | . | . | . | . | . | . | . | . | . | . | G | . | . | . | . | . | . | . |  | . | . | . | . | . | . | . |  | . | . | . | . | - | - | - | - | - | - | - |
| H20 | . | . | . | . | . | . | . | . | . | . | . | . | . | . | . | . | . | . | . | . | . |  | A | . | . | . | . | . | . |  | . | . | . | . | . | . | . | . | . | . | . |
| H21 | . | . | . | . | . | . | . | . | . | . | . | . | . | . | . | . | . | . | . | . | . |  | . | . | . | . | . | . | . |  | . | . | . | . | . | . | . | . | . | . | . |
| H22 | . | . | . | . | . | . | . | . | . | . | . | . | . | . | T | . | . | . | . | . | . |  | . | . | . | . | . | . | . |  | . | . | . | . | . | . | . | . | . | . | . |
| H23 | . | . | . | G | . | . | . | . | . | . | . | . | G | . | . | . | A | . | . | . | . |  | . | . | . | . | . | . | . |  | . | . | . | . | . | . | . | . | . | . | . |
| H24 | . | . | . | . | . | . | . | . | . | . | . | . | . | . | . | G | . | . | . | . | . |  | . | . | . | . | . | . | . |  | . | . | . | . | - | - | - | - | - | - | - |
| H25 | . | . | . | . | . | . | . | . | . | . | . | . | . | . | . | G | . | . | . | . | . |  | A | . | . | . | . | . | . |  | . | . | . | . | - | - | - | - | - | - | - |
| H26 | . | . | . | . | . | . | . | . | . | . | . | . | . | . | . | . | . | . | . | . | . |  | . | . | . | . | . | . | . |  | . | . | . | . | . | . | . | . | . | . | . |
| H27 | . | . | . | . | . | . | . | . | . | . | . | . | . | . | . | . | . | . | . | . | . |  | . | . | . | . | . | . | . |  | . | . | . | . | . | . | . | . | . | . | . |
| H28 | . | . | . | . | . | . | . | . | . | . | . | . | . | . | . | G | . | . | . | . | . |  | . | . | . | . | . | . | . |  | . | . | . | . | - | - | - | - | - | - | - |
| H29 | . | . | . | . | . | . | . | . | . | . | . | T | . | . | . | . | . | . | G | . | . |  | . | . | . | . | . | . | . |  | . | . | . | . | . | . | . | . | . | . | . |
| H30 | . | . | . | . | . | . | . | . | . | . | . | . | . | . | . | . | . | . | . | . | T |  | . | . | . | . | . | . | . |  | . | . | . | . | . | . | . | . | . | . | . |
| H31 | . | . | . | . | . | . | . | . | . | . | . | T | . | . | . | . | . | . | G | . | . |  | . | . | . | . | . | . | . |  | . | . | . | . | . | . | . | . | . | . | . |
| H32 | T | T | . | . | . | C | . | . | . | . | . | . | G | . | . | . | A | . | . | . | . |  | . | . | T | . | . | C | A |  | . | . | . | . | . | . | . | . | . | . | . |
| H33 | T | T | . | . | . | C | . | . | . | . | . | . | G | . | . | . | A | . | . | . | . |  | . | . | T | . | . | C | A |  | . | . | . | . | . | . | . | . | . | . | . |
| H34 | T | T | . | . | . | C | . | . | . | . | . | . | T | . | . | . | A | . | . | . | . |  | . | . | T | . | . | C | A |  | . | . | . | . | . | . | . | . | . | . | . |
| H35 | T | T | . | . | . | C | . | . | . | . | C | . | G | . | . | . | A | . | . | . | . |  | . | . | T | . | . | C | . |  | . | . | . | . | . | . | . | . | . | . | . |
| H36 | T | T | . | . | . | C | . | . | . | . | . | . | G | . | . | . | A | . | . | . | . |  | . | . | T | . | . | C | A |  | . | . | . | . | . | . | . | . | . | . | . |
| H37 | T | T | . | . | . | C | . | . | C | . | . | . | G | . | . | . | A | . | . | . | . |  | . | . | T | . | . | C | A |  | . | . | . | . | . | . | . | . | . | . | . |
| H38 | T | T | T | . | . | C | . | . | . | . | . | . | G | . | . | . | A | . | . | . | . |  | . | . | T | . | . | C | A |  | . | . | . | . | . | . | . | . | . | . | . |
| H39 | T | T | . | . | . | C | . | . | . | . | . | . | G | . | . | . | A | . | . | . | . |  | . | . | T | . | . | C | A |  | . | . | . | . | . | . | . | . | . | . | . |
| H40 | T | T | . | . | . | C | . | . | . | . | . | . | G | . | . | . | A | . | . | . | . |  | . | G | T | . | . | C | A |  | . | . | . | . | . | . | . | . | . | . | . |
| H41 | T | T | . | . | . | C | . | . | . | . | . | . | G | . | . | . | A | . | . | . | . |  | . | . | T | . | . | C | . |  | . | . | . | . | . | . | . | . | . | . | . |
| H42 | T | T | . | . | . | C | . | . | . | . | . | . | G | . | . | . | A | . | . | . | . |  | . | . | T | . | . | C | . |  | . | . | . | . | . | . | . | . | . | . | . |
| H43 | T | T | . | . | . | C | . | . | . | . | . | . | G | . | . | . | A | . | . | . | . |  | . | . | T | . | . | C | . |  | . | . | . | . | . | . | . | . | . | . | . |
| H44 | T | T | . | . | . | C | . | . | . | . | . | . | G | . | . | . | A | . | . | . | . |  | . | . | T | . | . | C | . |  | . | . | . | . | . | . | . | . | . | . | . |
| H45 | T | T | . | . | C | C | . | . | . | . | . | . | T | . | . | . | A | . | . | . | . |  | . | . | T | . | . | C | A |  | . | . | . | . | . | . | . | . | . | . | . |
| H46 | T | T | . | . | . | C | . | . | . | . | . | . | G | . | . | . | A | G | . | . | . |  | . | . | T | . | . | C | . |  | . | . | . | . | . | . | . | . | . | . | . |
| H47 | T | T | . | . | . | C | . | . | . | . | . | . | G | . | . | . | A | . | . | . | . |  | . | . | . | . | . | C | . |  | . | . | . | . | . | . | . | . | . | . | . |
| H48 | T | T | . | . | . | C | . | . | . | . | . | . | G | . | . | . | A | . | . | G | . |  | . | . | T | . | . | C | . |  | . | . | . | . | . | . | . | . | . | . | . |
| H49 | T | T | . | . | . | C | . | . | . | . | . | . | G | . | . | . | A | . | . | . | . |  | . | . | T | . | . | C | . |  | . | A | . | . | . | . | . | . | . | . | . |
| H50 | T | T | . | . | . | C | . | . | . | . | . | . | G | . | . | . | A | . | . | . | . |  | . | . | T | . | . | C | . |  | . | . | . | . | . | . | . | . | . | . | . |
| H51 | T | T | . | . | . | C | . | . | . | . | . | . | T | . | . | . | A | . | . | . | . |  | . | . | T | . | . | C | A |  | . | . | . | . | . | . | . | . | . | . | . |
| H52 | T | T | . | . | . | C | . | . | . | . | . | . | G | . | . | . | A | . | . | . | . |  | . | . | T | . | . | C | . |  | . | . | . | . | . | . | . | . | . | . | . |
| H53 | . | . | . | . | . | . | . | . | . | . | . | . | G | . | . | . | A | . | . | . | . |  | . | . | . | A | A | . | . |  | . | . | G | . | . | . | . | . | . | . | . |
| H54 | . | . | . | . | . | . | . | . | . | . | . | . | G | . | . | . | A | . | . | . | . |  | . | . | . | A | A | . | . |  | . | . | G | . | . | . | . | . | . | . | . |
| H55 | . | . | . | . | . | . | . | . | . | T | . | . | G | . | . | . | A | . | . | . | . |  | . | . | . | A | A | . | . |  | . | . | G | . | . | . | . | . | . | . | . |

| **cp -DNA Hap- loty- pes** | ***trn*S *- trn*G** | | | | | | | | | | | | | | | | | | | | | | | | | | | | | | | | | | | | | | |
| --- | --- | --- | --- | --- | --- | --- | --- | --- | --- | --- | --- | --- | --- | --- | --- | --- | --- | --- | --- | --- | --- | --- | --- | --- | --- | --- | --- | --- | --- | --- | --- | --- | --- | --- | --- | --- | --- | --- | --- |
|  | 1 | 1 | 1 | 1 | 1 | 1 | 1 | 1 | 1 | 1 | 1 | 1 | 1 | 1 | 1 | 1 | 1 | 1 | 1 | 1 | 1 | 1 | 1 | 1 | 1 | 1 | 1 | 1 | 1 | 1 | 1 | 1 | 1 | 1 | 1 | 1 | 1 | 1 | 1 |
|  | 4 | 4 | 4 | 4 | 4 | 4 | 4 | 4 | 4 | 4 | 4 | 4 | 4 | 4 | 4 | 4 | 4 | 4 | 5 | 5 | 5 | 5 | 5 | 5 | 5 | 5 | 5 | 5 | 5 | 5 | 5 | 5 | 5 | 5 | 5 | 5 | 5 | 5 | 5 |
|  | 5 | 5 | 5 | 5 | 5 | 5 | 5 | 6 | 6 | 6 | 6 | 6 | 8 | 8 | 8 | 8 | 8 | 9 | 0 | 3 | 3 | 3 | 3 | 3 | 3 | 3 | 4 | 4 | 5 | 5 | 5 | 5 | 5 | 5 | 5 | 5 | 5 | 5 | 7 |
|  | 3 | 4 | 5 | 6 | 7 | 8 | 9 | 0 | 2 | 3 | 4 | 5 | 1 | 2 | 3 | 4 | 9 | 3 | 8 | 3 | 4 | 5 | 6 | 7 | 8 | 9 | 8 | 9 | 0 | 1 | 2 | 3 | 4 | 5 | 6 | 7 | 8 | 9 | 8 |
| H1 | - | - | - | - | - | - | - | T | - | - | - | - | - | - | - | - | G | G | A | - | - | - | - | - | - | - | - | - | - | - | - | - | - | - | - | - | - | G | C |
| H2 | A | A | A | A | - | - | T | T | - | - | - | - | - | - | - | - | . | . | . | - | - | - | - | - | - | - | - | - | - | - | - | - | - | - | - | - | - | G | . |
| H3 | . | . | . | . | A | - | T | T | - | - | - | - | - | - | - | - | . | . | . | - | - | - | - | - | - | - | - | - | - | - | - | - | - | - | - | - | - | G | . |
| H4 | . | . | . | . | A | - | T | T | - | - | - | - | A | - | - | - | . | . | . | - | - | - | - | - | - | - | - | - | - | - | - | - | - | - | - | - | - | G | . |
| H5 | . | . | . | . | - | - | T | T | - | - | - | - | - | - | - | - | . | . | . | - | - | - | - | - | - | - | - | - | - | - | - | - | - | - | - | - | - | G | . |
| H6 | . | . | . | . | A | - | T | T | - | - | - | - | - | - | - | - | . | . | . | - | - | - | - | - | - | - | T | A | T | C | T | A | A | T | A | A | G | G | . |
| H7 | . | . | . | . | - | - | T | T | - | - | - | - | - | - | - | - | . | . | . | - | - | - | - | - | - | - | - | - | - | - | - | - | - | - | - | - | - | G | . |
| H8 | . | . | . | . | A | - | T | T | - | - | - | - | - | - | - | - | . | . | . | - | - | - | - | - | - | - | - | - | - | - | - | - | - | - | - | - | - | G | . |
| H9 | . | . | . | . | A | - | T | T | - | - | - | - | - | - | - | - | . | . | . | - | - | - | - | - | - | - | - | - | - | - | - | - | - | - | - | - | - | G | . |
| H10 | . | . | . | - | - | - | T | T | - | - | - | - | - | - | - | - | . | . | . | - | - | - | - | - | - | - | - | - | - | - | - | - | - | - | - | - | - | G | . |
| H11 | . | . | . | - | - | - | T | T | - | - | - | - | - | - | - | - | . | . | . | - | - | - | - | - | - | - | - | - | - | - | - | - | - | - | - | - | - | G | . |
| H12 | - | - | - | - | - | - | - | T | - | - | - | - | - | - | - | - | . | . | . | - | - | - | - | - | - | - | - | - | - | - | - | - | - | - | - | - | - | G | . |
| H13 | . | . | . | - | - | - | T | T | - | - | - | - | - | - | - | - | . | . | . | - | - | - | - | - | - | - | - | - | - | - | - | - | - | - | - | - | - | G | . |
| H14 | . | . | . | . | A | - | T | T | - | - | - | - | A | A | A | - | . | . | . | - | - | - | - | - | - | - | - | - | - | - | - | - | - | - | - | - | - | . | . |
| H15 | . | . | . | . | A | - | T | T | - | - | - | - | A | A | A | - | . | . | . | - | - | - | - | - | - | - | - | - | - | - | - | - | - | - | - | - | - | . | . |
| H16 | . | . | . | . | A | - | T | T | - | - | - | - | A | A | A | - | . | . | . | - | - | - | - | - | - | - | - | - | - | - | - | - | - | - | - | - | - | . | . |
| H17 | . | . | . | . | A | - | T | T | - | - | - | - | - | - | - | - | . | . | . | - | - | - | - | - | - | - | - | - | - | - | - | - | - | - | - | - | - | G | . |
| H18 | . | . | . | . | - | - | T | T | - | - | - | - | - | - | - | - | . | . | . | - | - | - | - | - | - | - | - | - | - | - | - | - | - | - | - | - | - | G | . |
| H19 | - | - | - | - | - | - | - | - | - | - | - | - | - | - | - | - | . | . | . | - | - | - | - | - | - | - | - | - | - | - | - | - | - | - | - | - | - | G | . |
| H20 | . | . | . | . | A | - | T | T | - | - | - | - | - | - | - | - | . | . | . | - | - | - | - | - | - | - | - | - | - | - | - | - | - | - | - | - | - | G | . |
| H21 | . | . | . | . | - | - | T | T | - | - | - | - | A | - | - | - | . | . | . | - | - | - | - | - | - | - | - | - | - | - | - | - | - | - | - | - | - | G | . |
| H22 | . | . | . | . | A | - | T | T | - | - | - | - | - | - | - | - | . | . | . | - | - | - | - | - | - | - | T | A | T | C | T | A | A | T | A | A | G | . | . |
| H23 | . | . | . | . | - | - | T | T | - | - | - | - | A | A | A | A | A | A | . | - | - | - | - | - | - | - | - | - | - | - | - | - | - | - | - | - | - | . | . |
| H24 | - | - | - | - | - | - | - | - | - | - | - | - | - | - | - | - | . | . | . | - | - | - | - | - | - | - | - | - | - | - | - | - | - | - | - | - | - | G | . |
| H25 | - | - | - | - | - | - | - | - | - | - | - | - | - | - | - | - | . | . | . | - | - | - | - | - | - | - | - | - | - | - | - | - | - | - | - | - | - | G | . |
| H26 | . | . | . | . | A | - | T | T | - | - | - | - | - | - | - | - | . | . | . | - | - | - | - | - | - | - | T | A | T | C | T | A | A | T | A | A | G | G | . |
| H27 | . | . | - | - | - | - | T | T | - | - | - | - | - | - | - | - | . | . | . | - | - | - | - | - | - | - | - | - | - | - | - | - | - | - | - | - | - | G | . |
| H28 | - | - | - | - | - | - | - | - | - | - | - | - | - | - | - | - | . | . | . | C | T | A | A | T | A | T | - | - | - | - | - | - | - | - | - | - | - | G | . |
| H29 | . | . | . | . | - | - | T | T | - | - | - | - | - | - | - | - | . | . | . | - | - | - | - | - | - | - | - | - | - | - | - | - | - | - | - | - | - | G | . |
| H30 | . | . | . | . | A | - | T | T | - | - | - | - | - | - | - | - | . | . | . | - | - | - | - | - | - | - | - | - | - | - | - | - | - | - | - | - | - | G | . |
| H31 | . | . | . | . | A | A | T | T | - | - | - | - | - | - | - | - | . | . | . | - | - | - | - | - | - | - | - | - | - | - | - | - | - | - | - | - | - | G | . |
| H32 | . | . | . | . | - | - | T | T | - | - | - | - | - | - | - | - | . | . | . | - | - | - | - | - | - | - | - | - | - | - | - | - | - | - | - | - | - | . | . |
| H33 | . | . | . | - | - | - | T | T | - | - | - | - | - | - | - | - | . | . | . | - | - | - | - | - | - | - | - | - | - | - | - | - | - | - | - | - | - | . | . |
| H34 | . | . | . | . | - | - | T | T | - | - | - | - | - | - | - | - | . | . | . | - | - | - | - | - | - | - | - | - | - | - | - | - | - | - | - | - | - | . | . |
| H35 | . | . | . | . | A | - | T | T | - | - | - | - | - | - | - | - | . | . | G | - | - | - | - | - | - | - | - | - | - | - | - | - | - | - | - | - | - | . | . |
| H36 | . | . | . | . | A | - | T | T | - | - | - | - | - | - | - | - | . | . | G | - | - | - | - | - | - | - | - | - | - | - | - | - | - | - | - | - | - | . | . |
| H37 | . | . | . | . | A | - | T | T | - | - | - | - | - | - | - | - | . | . | G | - | - | - | - | - | - | - | - | - | - | - | - | - | - | - | - | - | - | . | . |
| H38 | . | . | . | . | - | - | T | T | - | - | - | - | - | - | - | - | . | . | . | - | - | - | - | - | - | - | - | - | - | - | - | - | - | - | - | - | - | . | . |
| H39 | . | . | . | . | A | - | T | T | - | - | - | - | - | - | - | - | . | . | . | - | - | - | - | - | - | - | - | - | - | - | - | - | - | - | - | - | - | . | . |
| H40 | . | . | . | . | A | - | T | T | - | - | - | - | - | - | - | - | . | . | . | - | - | - | - | - | - | - | - | - | - | - | - | - | - | - | - | - | - | . | . |
| H41 | . | . | . | - | - | - | T | T | - | - | - | - | - | - | - | - | . | . | G | - | - | - | - | - | - | - | - | - | - | - | - | - | - | - | - | - | - | . | . |
| H42 | . | . | . | . | - | - | T | T | T | A | A | A | - | - | - | - | . | . | G | - | - | - | - | - | - | - | - | - | - | - | - | - | - | - | - | - | - | . | . |
| H43 | . | . | . | . | - | - | T | T | - | - | - | - | - | - | - | - | . | . | G | - | - | - | - | - | - | - | - | - | - | - | - | - | - | - | - | - | - | . | A |
| H44 | . | . | . | . | A | - | T | T | - | - | - | - | - | - | - | - | . | . | G | - | - | - | - | - | - | - | - | - | - | - | - | - | - | - | - | - | - | . | . |
| H45 | . | . | . | . | - | - | T | T | - | - | - | - | - | - | - | - | . | . | . | - | - | - | - | - | - | - | - | - | - | - | - | - | - | - | - | - | - | . | . |
| H46 | . | . | . | . | - | - | T | T | - | - | - | - | - | - | - | - | . | . | G | - | - | - | - | - | - | - | - | - | - | - | - | - | - | - | - | - | - | . | . |
| H47 | . | . | . | - | - | - | T | T | - | - | - | - | - | - | - | - | . | . | G | - | - | - | - | - | - | - | - | - | - | - | - | - | - | - | - | - | - | . | . |
| H48 | . | . | . | - | - | - | T | T | - | - | - | - | - | - | - | - | . | . | G | - | - | - | - | - | - | - | - | - | - | - | - | - | - | - | - | - | - | . | . |
| H49 | . | . | . | - | - | - | T | T | - | - | - | - | - | - | - | - | . | . | G | - | - | - | - | - | - | - | - | - | - | - | - | - | - | - | - | - | - | . | . |
| H50 | . | . | . | - | - | - | T | T | - | - | - | - | - | - | - | - | . | . | G | - | - | - | - | - | - | - | - | - | - | - | - | - | - | - | - | - | - | . | A |
| H51 | . | . | . | - | - | - | T | T | - | - | - | - | - | - | - | - | . | . | . | - | - | - | - | - | - | - | - | - | - | - | - | - | - | - | - | - | - | . | . |
| H52 | . | . | . | . | - | A | T | T | T | A | A | A | - | - | - | - | . | . | G | - | - | - | - | - | - | - | - | - | - | - | - | - | - | - | - | - | - | . | . |
| H53 | . | . | . | . | - | - | T | T | - | - | - | - | - | - | - | - | . | . | . | - | - | - | - | - | - | - | - | - | - | - | - | - | - | - | - | - | - | . | . |
| H54 | . | . | . | . | A | - | T | T | - | - | - | - | - | - | - | - | . | . | . | - | - | - | - | - | - | - | - | - | - | - | - | - | - | - | - | - | - | . | . |
| H55 | . | . | . | . | - | - | T | T | - | - | - | - | - | - | - | - | . | . | . | - | - | - | - | - | - | - | - | - | - | - | - | - | - | - | - | - | - | . | . |

The number of each mutation site is read vertically in the table above. “.” indicates a base pair according to the first horizontal line. Each letter indicates the mutation, except for the first letter. “-” indicates no base pair (gap).

**Table S4.** Variable sites in the aligned ITS sequences.

| **ITS Hap- loty- pes** | **ITS** | | | | | | | | | | | | | | | | | | | | | | | | | | | | | | | | |
| --- | --- | --- | --- | --- | --- | --- | --- | --- | --- | --- | --- | --- | --- | --- | --- | --- | --- | --- | --- | --- | --- | --- | --- | --- | --- | --- | --- | --- | --- | --- | --- | --- | --- |
|  | 0 | 0 | 0 | 0 | 0 | 0 | 0 | 0 | 0 | 0 | 0 | 0 | 0 | 0 | 0 | 0 | 0 | 0 | 0 | 0 | 0 | 0 | 1 | 1 | 1 | 1 | 1 | 1 | 1 | 1 | 1 | 1 | 1 |
|  | 0 | 0 | 1 | 1 | 1 | 2 | 2 | 3 | 3 | 4 | 4 | 4 | 4 | 5 | 5 | 6 | 6 | 7 | 7 | 8 | 8 | 9 | 0 | 1 | 4 | 4 | 4 | 4 | 6 | 6 | 6 | 7 | 8 |
|  | 5 | 6 | 1 | 4 | 9 | 4 | 8 | 5 | 6 | 0 | 2 | 3 | 9 | 4 | 8 | 1 | 9 | 6 | 7 | 0 | 5 | 9 | 1 | 5 | 0 | 1 | 6 | 9 | 0 | 5 | 9 | 0 | 1 |
| H1 | C | C | C | C | - | G | G | C | C | T | C | G | C | A | G | C | G | A | T | A | C | C | C | G | G | C | C | C | G | C | C | C | C |
| H2 | . | . | . | . | - | . | . | . | . | . | T | . | . | . | . | . | . | . | . | A | . | . | . | . | . | . | . | . | . | . | C | . | . |
| H3 | . | . | . | . | - | . | . | . | . | . | T | . | . | . | . | . | . | . | . | A | . | . | . | . | T | . | . | . | . | . | C | . | . |
| H4 | . | . | . | . | - | . | . | . | . | . | . | . | . | . | . | . | . | . | . | A | . | . | . | . | . | . | . | . | . | . | C | . | . |
| H5 | . | . | . | . | - | . | . | . | . | . | . | . | . | . | . | T | . | . | . | A | . | . | . | . | T | . | . | . | . | . | C | . | . |
| H6 | . | . | . | . | - | . | . | . | . | . | . | . | . | . | . | T | . | . | . | A | . | . | . | . | . | . | . | . | . | . | C | . | . |
| H7 | . | . | . | . | - | . | . | . | . | . | . | . | . | . | . | T | . | . | . | A | . | . | . | . | . | . | . | . | . | . | C | . | . |
| H8 | . | . | . | . | - | . | . | . | . | . | . | . | . | . | . | . | . | . | . | A | . | . | . | . | . | . | . | . | . | . | C | . | . |
| H9 | . | . | . | . | - | . | . | . | . | C | . | . | . | . | . | T | . | . | . | A | . | . | . | . | . | . | . | . | . | . | C | . | . |
| H10 | . | . | . | . | - | . | . | . | . | C | . | . | . | . | . | . | . | . | . | A | . | . | . | . | . | . | . | . | . | . | C | . | . |
| H11 | . | . | . | . | - | . | . | . | . | C | . | . | . | . | . | . | . | . | . | A | . | . | . | . | . | . | . | . | . | . | C | . | . |
| H12 | . | . | . | . | - | . | . | . | . | . | . | . | . | . | . | T | . | . | . | A | . | . | . | . | . | . | . | . | . | . | C | . | . |
| H13 | . | . | . | . | - | . | . | . | . | . | . | . | . | . | . | . | . | . | . | A | . | . | . | . | . | . | . | . | . | . | C | . | . |
| H14 | . | . | . | . | - | . | . | . | . | . | . | . | . | . | . | T | . | . | . | A | . | . | . | . | T | . | . | . | . | . | C | . | . |
| H15 | . | . | . | . | - | . | . | . | . | . | . | . | . | . | . | T | . | . | . | A | . | . | . | . | . | . | . | . | . | . | C | . | . |
| H16 | . | . | . | . | - | . | . | . | . | . | . | . | . | . | . | T | . | . | . | A | . | . | . | . | . | . | . | . | . | . | C | . | . |
| H17 | . | . | . | . | T | . | . | . | . | . | . | . | . | . | . | T | . | . | . | A | . | . | . | . | . | . | . | . | . | . | C | . | . |
| H18 | . | . | . | . | T | . | . | . | . | . | . | . | . | . | . | T | . | . | . | A | . | . | . | . | . | . | . | . | . | . | C | . | . |
| H19 | . | . | . | . | - | . | . | . | . | . | . | . | . | . | . | T | . | . | . | A | . | . | . | . | . | . | . | . | . | . | C | . | . |
| H20 | . | . | . | . | - | . | . | . | . | . | . | . | . | . | . | T | . | . | . | A | . | . | . | . | . | . | . | . | . | . | C | . | . |
| H21 | . | . | . | . | - | . | . | . | . | G | . | . | . | . | . | . | . | . | . | A | . | . | . | . | . | . | . | . | . | . | C | . | . |
| H22 | . | . | . | T | - | . | C | T | . | . | . | . | . | C | A | . | . | . | C | G | T | . | . | C | . | . | T | . | . | A | T | . | . |
| H23 | . | . | . | T | - | . | C | T | . | . | . | . | . | C | A | . | A | . | C | . | T | . | . | C | . | . | T | T | . | . | . | . | . |
| H24 | . | . | . | T | - | . | C | T | . | . | . | . | . | C | . | . | . | T | C | . | T | . | T | T | . | . | T | . | . | . | . | T | . |
| H25 | . | . | . | T | - | . | C | T | . | . | . | . | . | C | . | . | . | T | C | . | T | . | . | T | . | . | T | . | . | . | . | T | . |
| H26 | . | . | . | T | - | . | C | T | . | . | . | . | . | C | . | . | . | . | C | . | T | . | . | T | . | . | T | . | . | . | . | T | . |
| H27 | . | . | . | T | - | . | C | T | . | . | . | . | . | C | A | . | . | . | C | . | T | . | . | C | . | . | T | . | T | A | . | . | . |
| H28 | . | . | . | T | - | . | C | T | . | . | . | . | . | C | A | . | . | . | C | . | T | . | . | C | . | . | T | . | T | A | . | . | . |
| H29 | . | . | . | T | - | . | C | T | . | . | . | . | . | C | A | . | . | . | C | . | T | . | . | C | . | . | T | . | . | A | . | . | . |
| H30 | . | . | . | T | - | . | C | T | . | . | . | . | . | C | A | . | . | . | C | . | T | . | . | C | . | . | T | . | . | A | . | . | . |
| H31 | . | . | . | T | - | . | C | T | . | . | . | . | . | C | . | . | . | . | C | . | T | . | . | C | . | . | T | . | . | . | . | T | . |
| H32 | . | . | . | T | - | . | C | T | . | . | . | . | . | C | A | . | . | . | C | . | T | . | . | C | . | . | T | . | . | A | . | . | . |
| H33 | . | . | . | T | - | . | C | T | . | . | . | . | . | C | A | . | . | . | C | . | T | . | . | C | . | . | T | . | T | A | . | . | . |
| H34 | . | . | . | T | - | . | C | T | . | . | . | . | . | C | A | . | . | . | C | . | T | . | . | C | . | . | T | . | . | A | . | . | . |
| H35 | . | . | . | T | - | . | C | T | . | . | . | . | . | C | A | . | . | . | C | . | T | . | . | C | . | . | T | . | . | A | . | . | . |
| H36 | . | . | . | T | - | . | C | T | . | . | . | . | A | C | . | . | . | . | C | . | T | . | . | T | . | . | T | . | . | . | . | T | . |
| H37 | . | . | . | T | - | . | C | T | . | . | . | . | A | C | . | . | . | T | C | . | T | . | . | T | . | . | T | . | . | . | . | T | . |
| H38 | . | . | . | T | - | . | C | T | . | . | . | . | A | C | . | . | . | . | C | . | T | T | . | T | . | . | T | . | . | . | . | T | . |
| H39 | . | . | . | T | - | . | C | T | . | . | . | . | A | C | . | . | . | . | C | . | T | . | . | T | . | . | T | . | . | . | . | T | . |
| H40 | . | . | . | T | - | . | C | T | . | . | . | . | . | C | . | . | . | . | C | . | T | . | . | C | . | . | T | . | . | A | . | . | . |
| H41 | . | . | . | T | - | . | C | T | . | . | . | . | . | C | A | . | . | . | C | . | T | . | . | T | . | . | T | . | . | A | . | . | . |
| H42 | . | . | . | T | - | . | C | T | . | . | . | . | A | C | . | . | . | T | C | . | T | . | . | T | . | . | T | . | . | . | . | T | . |
| H43 | . | T | . | . | - | T | . | . | . | C | . | A | . | . | . | . | . | . | . | . | . | . | . | . | . | T | . | . | . | . | C | . | . |
| H44 | . | T | . | . | - | T | . | . | . | C | . | A | . | . | . | . | . | . | . | . | . | . | . | . | . | T | . | . | . | . | . | . | . |
| H45 | . | T | . | . | - | T | . | . | . | C | . | A | . | . | . | . | . | . | . | . | . | . | . | . | . | T | . | . | . | . | . | . | . |
| H46 | . | T | . | . | - | T | . | . | . | C | . | A | . | . | . | . | . | . | . | . | . | . | . | . | . | T | . | . | . | . | C | . | . |
| H47 | . | T | . | . | - | T | . | . | . | C | . | A | . | . | . | . | . | . | . | . | . | . | . | . | . | T | . | . | . | . | . | . | . |
| H48 | T | . | G | . | - | . | . | . | T | . | . | . | T | . | . | . | . | . | C | A | . | . | . | . | . | . | . | T | . | . | C | . | T |

| **TS Hap- loty- pes** | **ITS** | | | | | | | | | | | | | | | | | | | | | | | | | | | | | | | | |
| --- | --- | --- | --- | --- | --- | --- | --- | --- | --- | --- | --- | --- | --- | --- | --- | --- | --- | --- | --- | --- | --- | --- | --- | --- | --- | --- | --- | --- | --- | --- | --- | --- | --- |
|  | 2 | 2 | 3 | 3 | 3 | 3 | 3 | 3 | 3 | 3 | 3 | 3 | 3 | 3 | 4 | 4 | 4 | 4 | 4 | 4 | 4 | 4 | 4 | 4 | 4 | 4 | 4 | 5 | 5 | 5 | 5 | 5 | 5 |
|  | 0 | 5 | 0 | 0 | 3 | 3 | 6 | 6 | 7 | 7 | 8 | 8 | 8 | 9 | 1 | 2 | 2 | 2 | 2 | 3 | 5 | 5 | 6 | 8 | 8 | 9 | 9 | 0 | 2 | 2 | 2 | 4 | 5 |
|  | 3 | 8 | 2 | 7 | 3 | 8 | 4 | 9 | 5 | 9 | 0 | 1 | 5 | 9 | 8 | 0 | 1 | 2 | 3 | 6 | 4 | 8 | 0 | 1 | 3 | 2 | 7 | 0 | 0 | 6 | 7 | 9 | 0 |
| H1 | C | G | T | T | T | C | C | T | G | A | G | C | T | C | G | C | G | T | G | G | A | C | T | G | C | C | C | G | G | C | G | C | C |
| H2 | . | . | T | T | . | . | . | T | . | . | . | . | . | C | . | . | . | T | . | . | . | . | . | . | . | . | . | G | G | . | . | C | . |
| H3 | . | . | T | T | . | . | . | T | . | . | . | . | . | C | . | . | . | T | . | . | . | . | . | . | . | . | . | G | G | . | . | C | . |
| H4 | . | . | T | T | . | . | . | T | . | . | . | . | . | C | . | . | . | T | . | . | . | . | . | . | . | . | T | G | G | . | . | C | . |
| H5 | . | . | T | T | . | . | . | T | . | . | . | . | . | C | . | . | . | T | . | . | . | . | . | . | . | . | T | G | G | . | . | C | . |
| H6 | . | . | T | T | . | . | T | T | . | . | . | . | . | C | T | . | . | T | . | . | . | . | . | . | . | . | . | G | G | . | . | C | . |
| H7 | . | . | T | T | . | . | . | T | . | . | . | . | . | C | . | . | . | T | . | . | . | . | . | . | . | . | T | G | G | . | . | C | . |
| H8 | . | . | T | T | . | . | T | T | . | . | . | . | . | C | T | . | . | T | . | . | . | . | . | . | . | . | . | G | G | . | . | C | . |
| H9 | . | . | T | T | . | . | . | T | . | . | . | . | . | C | . | . | . | T | . | . | . | . | . | . | . | . | . | G | G | . | . | C | . |
| H10 | . | . | T | T | . | . | . | T | . | . | . | . | . | C | . | . | . | T | . | . | . | . | . | . | . | . | . | G | G | . | . | C | . |
| H11 | . | . | T | T | . | . | . | T | . | . | . | . | . | C | . | . | . | T | . | . | . | . | . | . | . | . | T | G | G | . | . | C | . |
| H12 | . | . | T | T | . | . | T | T | . | . | . | . | . | C | T | . | . | T | . | . | . | . | . | . | . | . | T | G | G | . | . | C | . |
| H13 | . | . | T | T | . | . | . | T | . | . | . | . | . | C | T | . | . | T | . | . | . | . | . | . | . | . | . | G | G | . | . | C | . |
| H14 | . | . | T | T | . | . | . | T | . | . | . | . | . | C | . | . | . | T | . | . | . | . | . | . | . | . | . | G | G | . | . | C | . |
| H15 | . | . | T | T | . | . | . | T | . | . | C | . | . | C | . | . | . | T | . | . | . | . | . | . | . | . | T | G | G | . | . | C | . |
| H16 | . | . | T | T | . | . | . | T | . | . | . | . | . | C | . | . | . | T | . | . | . | . | . | . | . | . | . | G | G | . | . | C | . |
| H17 | . | . | T | T | . | . | . | T | . | . | . | . | . | C | . | . | . | T | . | . | . | . | . | . | . | . | T | G | G | . | . | C | . |
| H18 | . | A | T | T | . | . | . | T | A | . | . | . | . | C | . | . | . | T | . | . | . | . | . | A | . | . | T | G | G | . | A | C | . |
| H19 | . | . | T | T | . | . | . | T | . | . | . | . | . | C | . | . | . | T | . | . | C | . | . | . | . | . | T | G | G | . | . | C | . |
| H20 | . | . | T | T | . | . | . | T | . | . | . | . | . | C | . | . | . | T | . | . | T | . | . | . | . | . | T | G | G | . | . | C | . |
| H21 | . | . | T | T | . | . | . | T | . | . | . | . | . | C | . | . | . | T | . | . | . | . | . | . | . | . | . | G | G | . | . | C | . |
| H22 | . | . | C | C | . | . | . | C | . | . | . | . | G | T | . | . | . | C | T | . | . | . | . | . | . | T | . | A | C | . | . | T | . |
| H23 | . | . | . | . | . | . | . | . | . | . | . | . | G | . | . | . | . | . | T | . | . | . | . | . | . | T | . | . | . | . | . | . | . |
| H24 | . | . | . | . | . | . | . | . | . | . | . | . | G | . | . | . | . | . | T | . | . | . | . | . | . | T | . | . | . | T | . | . | . |
| H25 | . | . | . | . | . | . | . | . | . | . | . | . | G | . | . | . | . | . | T | . | . | . | . | . | . | T | . | . | . | T | . | . | . |
| H26 | . | . | . | . | . | . | . | . | . | . | . | . | G | . | . | . | . | . | T | . | . | . | . | . | . | T | . | . | . | . | . | . | . |
| H27 | . | . | . | . | . | . | . | . | . | . | . | . | G | . | . | . | . | . | T | . | . | . | . | C | . | T | . | . | . | . | . | . | . |
| H28 | . | . | . | . | . | . | . | . | . | . | . | . | G | . | . | . | . | . | T | . | . | . | . | . | . | T | . | . | T | . | . | . | . |
| H29 | . | . | . | . | . | . | . | . | . | . | . | . | G | . | . | . | . | . | T | . | . | . | . | . | . | T | . | . | T | . | . | . | . |
| H30 | . | . | . | . | . | . | . | . | . | . | . | . | G | . | . | . | . | . | T | . | . | . | . | C | . | T | . | . | . | . | . | . | . |
| H31 | . | . | . | . | . | . | . | . | . | . | . | . | G | . | . | . | . | . | T | . | . | . | . | . | . | T | . | . | . | T | . | . | . |
| H32 | . | . | . | . | . | . | . | . | . | . | . | A | G | . | . | . | . | . | T | . | . | . | . | C | . | T | . | . | . | . | . | . | . |
| H33 | . | . | . | . | . | . | . | . | . | . | . | . | G | . | . | . | . | . | T | . | . | . | . | . | . | T | . | . | . | . | . | . | . |
| H34 | . | . | . | . | . | . | . | . | . | . | . | A | G | . | . | . | . | . | T | . | . | . | . | . | . | T | . | . | . | . | . | . | . |
| H35 | . | . | . | . | . | . | . | . | . | . | . | . | G | . | . | . | . | . | T | . | . | . | . | . | . | T | . | . | . | . | . | . | T |
| H36 | . | . | . | . | . | . | . | . | . | . | . | . | G | . | . | . | . | . | T | . | . | . | . | . | . | T | . | . | . | . | . | . | . |
| H37 | . | . | . | . | . | . | . | . | . | . | . | . | G | . | . | . | . | . | T | . | . | . | . | . | . | T | . | . | . | . | . | . | . |
| H38 | . | . | . | . | . | . | . | . | . | . | . | . | G | . | . | . | . | . | T | . | . | . | . | . | . | T | . | . | . | . | . | . | . |
| H39 | . | . | . | . | . | . | . | . | . | T | . | . | G | . | . | . | . | . | T | . | . | . | . | . | . | T | . | . | . | T | . | . | . |
| H40 | . | . | . | . | . | . | . | . | . | . | . | . | G | . | . | . | . | . | T | . | . | . | . | . | . | T | . | . | . | . | . | . | . |
| H41 | . | . | . | . | . | . | . | . | . | . | . | . | G | . | . | . | . | . | T | . | . | . | . | . | . | T | . | . | . | . | . | . | . |
| H42 | . | . | . | . | . | . | . | . | . | . | . | . | G | . | . | . | . | . | T | . | . | . | . | . | . | T | . | . | . | T | . | . | . |
| H43 | . | . | . | . | C | . | . | . | . | . | . | T | . | . | . | T | . | . | . | . | . | . | . | . | A | . | A | . | . | . | . | . | . |
| H44 | . | . | . | . | C | . | . | . | . | . | . | T | . | . | . | T | . | . | . | . | . | . | . | . | A | . | A | . | . | . | . | . | . |
| H45 | . | . | . | . | C | . | . | . | . | . | . | T | . | . | . | T | . | . | . | T | . | . | . | . | A | . | A | . | . | . | . | . | . |
| H46 | . | . | . | . | C | . | . | . | . | . | . | T | . | C | . | T | . | . | . | . | . | . | . | . | A | . | A | . | . | . | . | . | . |
| H47 | . | . | . | . | C | . | . | . | . | . | . | T | . | C | . | T | . | . | . | . | . | . | . | . | A | . | A | . | . | . | . | . | . |
| H48 | T | . | T | . | . | T | . | . | . | . | . | . | . | C | . | . | A | . | . | . | . | T | C | . | . | . | . | . | . | . | . | C | T |

The number of each mutation site is read vertically in the table above. “.” indicates a base pair according to the first horizontal line. Each letter indicates the mutation, except for the first letter. “-” indicates no base pair (gap).

**Table S5.** Analysis of molecular variance (AMOVA) within *Notopterygium* species based on cpDNA datasets.

| **Species Name** | **Source of variation** | **d.f.** | **SS** | **VC** | **PV (%)** | **Fixation indices** |
| --- | --- | --- | --- | --- | --- | --- |
| ***N. incisum*** | Among populations | 27 | 901.77 | 4.39045 | 81.96 | *F*_ST_ = 0.81964 |
|  | Within population | 180 | 173.9 | 0.96611 | 18.04 |  |
|  | Total | 207 | 1075.668 | 5.35656 |  |  |
| ***N. franchetii*** | Among populations | 28 | 96.887 | 0.50565 | 83.91 | *F*_ST_ = 0.83909 |
|  | Within population | 165 | 16.0 | 0.09697 | 16.09 |  |
|  | Total | 193 | 112.887 | 0.60262 |  |  |
| ***N. oviforme*** | Among populations | 12 | 167.381 | 1.74552 | 84.74 | *F*_ST_ = 0.84742 |
|  | Within population | 91 | 28.6 | 0.31429 | 15.26 |  |
|  | Total | 103 | 195.981 | 2.05981 |  |  |
| ***N. forrestii*** | Among populations | 3 | 8.1 | 0.26167 | 75.85 | *F*_ST_ = 0.75845 |
|  | Within population | 36 | 3 | 0.08333 | 24.15 |  |
|  | Total | 39 | 11.1 | 0.345 |  |  |
| **All Samples** | Among species | 3 | 1468.140 | 3.71604 | 58.04 | *F*_CT_ = 0.58040 |
|  | Among populations |  |  |  |  | *F*_ST_ = 0.92706 |
|  | Within species | 71 | 1175.082 | 2.21953 | 34.67 |  |
|  | Within population | 476 | 222.3 | 0.46702 | 7.29 | *F*_SC_ = 0.82616 |
|  | Total | 550 | 2865.523 | 6.40258 |  |  |

Abbreviations: d.f., degrees of freedom; SS, sum of squares; VC, variance components; PV, percentage variation; *F*_SC_, *F*_ST_, and *F*_CT_ are fixation indices.

**Table S6.** Analysis of molecular variance (AMOVA) within *Notopterygium* based on ITS datasets.

| **Group** | **Source of variation** | **d.f.** | **SS** | **VC** | **PV (%)** | **F.statistics** |
| --- | --- | --- | --- | --- | --- | --- |
| ***N. incisum*** | Among populations | 27 | 109.6 | 0.49680 | 63.90 | *F*_ST_ = 0.63898 |
|  | Within population | 186 | 52.208 | 0.28069 | 36.10 |  |
|  | Total | 213 | 161.808 | 0.77749 |  |  |
| ***N. franchetii*** | Among populations | 28 | 176.764 | 0.88816 | 80.13 | *F*_ST_ = 0.80132 |
|  | Within population | 171 | 37.656 | 0.22021 | 19.87 |  |
|  | Total | 199 | 214.420 | 1.10837 |  |  |
| ***N. oviforme*** | Among populations | 12 | 69.214 | 0.72597 | 95.11 | *F*_ST_ = 0.95111 |
|  | Within population | 92 | 3.433 | 0.03732 | 4.89 |  |
|  | Total | 104 | 72.648 | 0.76329 |  |  |
| ***N. forrestii*** | Among populations | - | - | - | - | *-* |
|  | Within population | - | - | - | - | - |
|  | Total | - | - | - | - | - |
| **All Samples** | Among species | 3 | 4247.946 | 10.95909 | 92.87 | *F*_CT_ = 0.92873 |
|  | Among populations |  |  |  |  | *F*_ST_ = 0.98383 |
|  | Within species | 71 | 356.349 | 0.65020 | 5.51 |  |
|  | Within population | 489 | 93.297 | 0.19079 | 1.62 | *F*_SC_ = 0.77313 |
|  | Total | 563 | 4697.592 | 11.80008 |  |  |

Abbreviations: d.f., degrees of freedom; SS, sum of squares; VC, variance components; PV, percentage variation; *F*_SC_, *F*_ST_, and *F*_CT_ are fixation indices.

**Table S7.** Ecological variables for *Notopterygium incisum* and *Notopterygium franchetii*.

| **Species** | **Variable** | **Percentage contribution** | **Permutation importance** | **Explanation** |
| --- | --- | --- | --- | --- |
| *N. incisum* | bio4 | 20 | 9.2 | Monthly mean temperature difference between day and night |
|  | bio19 | 13.3 | 19 | Day and night temperature difference and annual temperature difference ratio |
|  | bio15 | 13 | 18.3 | Temperature change in season |
|  | bio7 | 9.2 | 6.2 | Variation in rainfall |
|  | bio5 | 7.3 | 1.7 | Warmest average rainfall |
|  | bio18 | 6.7 | 5.7 | Lowest average monthly rainfall |
| *N. franchetii* | bio4 | 22.7 | 14.1 | Annual average temperature |
|  | bio19 | 13.8 | 22.3 | Seasonal temperature change |
|  | bio15 | 12.2 | 8.8 | Annual average rainfall |
|  | bio11 | 7.1 | 14 | Variation of rainfall |
|  | bio12 | 6.9 | 6.6 | Warmest average rainfall |
|  | bio18 | 5.2 | 4.1 | Lowest average monthly rainfall |


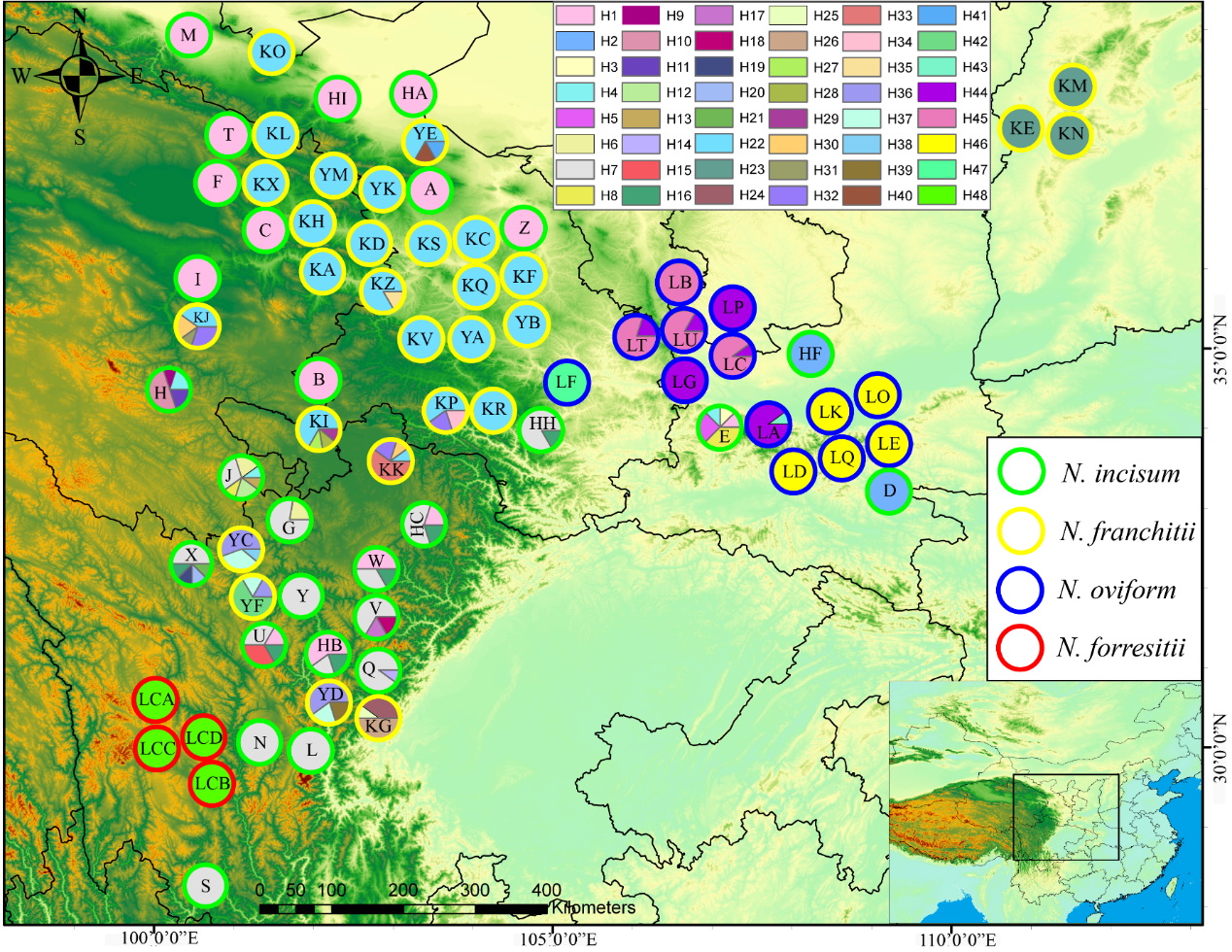


**Figure S1**

**
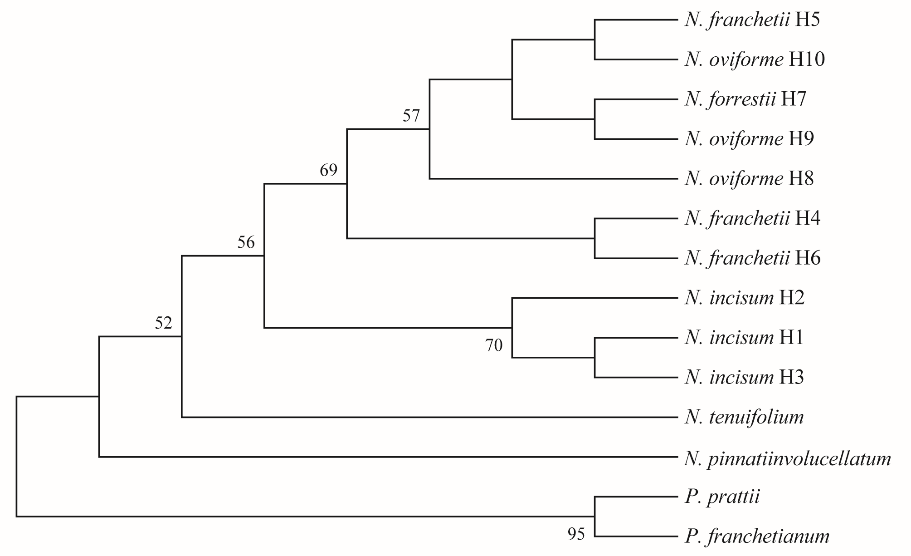
**

**Figure S2**


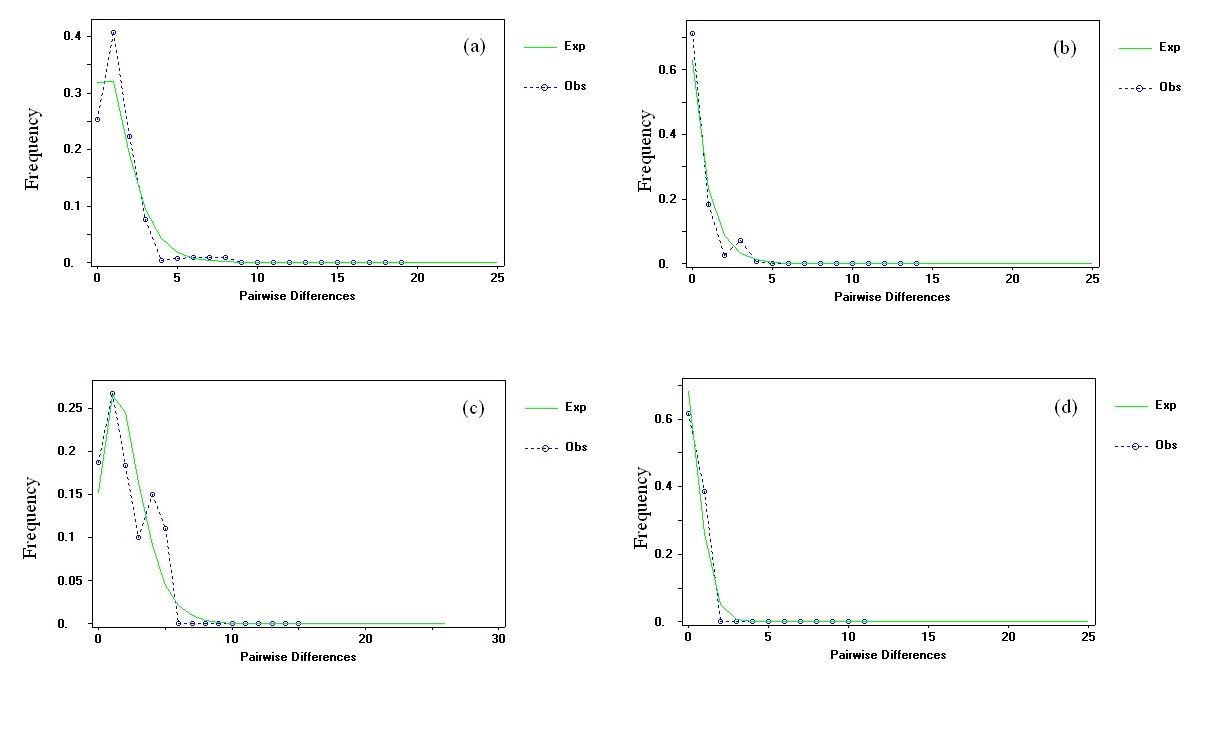


**Figure S3**
